# Substrate Binding Switches the Conformation at the Lynchpin Site in the Substrate-Binding Domain of Human Hsp70 to Enable Allosteric Interdomain Communication

**DOI:** 10.3390/molecules23030528

**Published:** 2018-02-27

**Authors:** Kohei Umehara, Miho Hoshikawa, Naoya Tochio, Shin-ichi Tate

**Affiliations:** 1 Department of Mathematical and Life Sciences, School of Science, Hiroshima University, 1-3-1 Kagamiyama, Higashi-Hiroshima 739-8526, Japan; m161409@hiroshima-u.ac.jp (K.U.); miho.hoshikawa.0925@gmail.com (M.H.); 2 Research Center for the Mathematics on Chromatin Live Dynamics (RcMcD), Hiroshima University, 1-3-1 Kagamiyama, Higashi-Hiroshima 739-8526, Japan; naoya-tochio@hiroshima-u.ac.jp or tochio@pharm.teikyo-u.ac.jp

**Keywords:** Hsp70, SBD, αLid, NMR

## Abstract

The stress-induced 70 kDa heat shock protein (Hsp70) functions as a molecular chaperone to maintain protein homeostasis. Hsp70 contains an N-terminal ATPase domain (NBD) and a C-terminal substrate-binding domain (SBD). The SBD is divided into the β subdomain containing the substrate-binding site (βSBD) and the α-helical subdomain (αLid) that covers the βSBD. In this report, the solution structures of two different forms of the SBD from human Hsp70 were solved. One structure shows the αLid bound to the substrate-binding site intramolecularly, whereas this intramolecular binding mode is absent in the other structure solved. Structural comparison of the two SBDs from Hsp70 revealed that client-peptide binding rearranges residues at the interdomain contact site, which impairs interdomain contact between the SBD and the NBD. Peptide binding also disrupted the inter-subdomain interaction connecting the αLid to the βSBD, which allows the binding of the αLid to the NBD. The results provide a mechanism for interdomain communication upon substrate binding from the SBD to the NBD via the lynchpin site in the βSBD of human Hsp70. In comparison to the bacterial ortholog, DnaK, some remarkable differences in the allosteric signal propagation among residues within the Hsp70 SBD exist.

## 1. Introduction

Heat shock protein 70 (Hsp70) molecular chaperones engage in a wide range of cellular processes integral to protein homeostasis [1,2]. These ATP-dependent chaperones monitor various protein-folding processes in cells through their promiscuous binding to proteins in unfolded, misfolded, or aggregated states but not to their folded counterparts [3,4]. The human genome encodes at least eight paralogs of Hsp70 [1,5]. Some human Hsp70 family members are functionally specialized to particular cellular compartments, including the cytosol, endoplasmic reticulum and mitochondria. Some Hsp70 members are expressed constitutively, whereas others are stress-induced [5]. The stress-inducible human Hsp70 (also called HSPA1A) is of particular interest because of its potential role in keeping cancer cells alive by preventing the formation of toxic protein aggregates that frequently occur in cancer cells [6]; cancer cells contain proteins with missense mutations that reduce their structural stability and thus have higher propensity to aggregate. Thus, cancer cells favor the presence and activity of Hsp70s for survival. This is observed for stress-induced Hsp70, which is upregulated in most cancer cells, although it is not essential for cell viability [6,7,8]. Thus, inhibitors of the activity of this Hsp70 are anticipated to be anti-tumor therapeutics [9,10,11,12,13]. 

Hsp70 members are evolutionally conserved, and their orthologs are found in both prokaryotes and eukaryotes [14]. The stress-induced human Hsp70 has an ortholog in *Escherichia coli* named DnaK [1,2]. Among the Hsp70 family of proteins, extensive structural studies of the DnaK have provided atomic level insights into the function of this protein and how this function is regulated, and these observations are mostly valid in human Hsp70 because of the high sequence similarity between human Hsp70 and DnaK [1,5,15]. 

**Figure 1:**
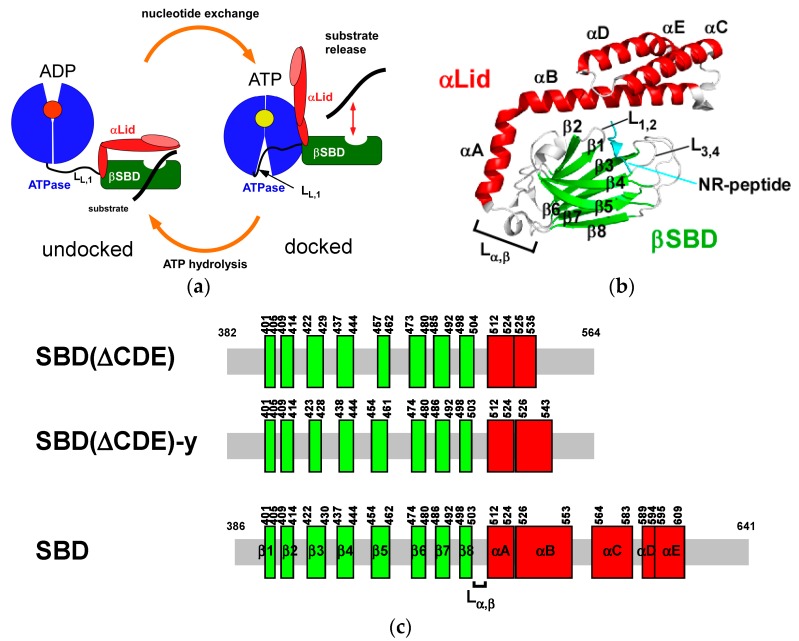
Hsp70 chaperone cycle and the domain architecture of the SBD. (**a**) Schematic drawing of the Hsp70 chaperone cycle; (**b**) The crystal structure of the SBD in human Hsp70 (HSPA1A) complexed with the NR-peptide harboring the sequence NRLLLTG (PDB ID: 4PO2) [15]; (**c**) The architecture of the SBD constructs used in this work. Secondary structures depicted for SBD(∆CDE) and SBD(∆CDE)-y were determined by NMR. SBD depicts the full-length sequence of the substrate-binding domain with the secondary structure taken from the crystal structure of the substrate-bound SBD in Hsp70 (PDB ID: 4PO2).

Hsp70 is composed of two domains: an N-terminal nucleotide-binding domain (NBD, 1–383) that has ATPase activity and a C-terminal peptide substrate-binding domain (SBD, 397–641). The two domains in Hsp70 are connected by the conserved hydrophobic linker (L_L,1_: ^384^KSENVQDLLLLDV^396^) [1,2] (Figure 1a). The action of the two domains in Hsp70 is allosterically coupled [16]. In the ADP bound form, the SBD binds to substrates with high affinity, whereas the NBD in the ATP bound state reduces substrate affinity of the SBD to accelerate substrate exchange (Figure 1a). 

The SBD binds to exposed hydrophobic stretches of misfolded or unfolded client proteins, and this domain contains a β-sandwich fold (βSBD: 397–503) and an α-helical lid (αLid: 510–614) followed by a disordered region (615–641) (Figure 1b). The structures of DnaK in the ADP-bound and ATP-bound forms show distinct arrangements of the four structural parts (the NBD, inter domain linker (L_L,1_), βSBD and αLid) in response to ATP hydrolysis (Figure 1a) [17,18,19,20,21]. In the ADP-bound (domain-undocked) state, the two domains behave independently with the interdomain linker released from the NBD and the αLid sitting across the βSBD to stabilize the substrate bound state [17,20]. ATP binding induces L_L,1_ binding to the NBD to facilitate the interaction with NBD and SBD with flipping of the αLid from the SBD to the NBD (Figure 1a) [18,19].

In the Hsp70 chaperone cycle, there is two-way allosteric control to link NBD and SBD functionally through their physical contact. The basal ATPase activity in NBD is low, and ATP hydrolysis is the rate-limiting step in the chaperone cycle (Figure 1a). Substrate binding to the SBD elevates ATPase activity to promote ATP hydrolysis and drive the progression of the chaperone cycle by converting the NBD to the ADP bound form (SBD to NBD control), which conversely enhances substrate binding to the SBD by detachment of the domains from each other (NBD to SBD control) (Figure 1a) [2]. 

The crystal structure of human Hsp70 in complex with a substrate peptide showed that the αLid covers the substrate binding cleft formed by loops L_1,2_ and L_3,4_ in βSBD (PDB ID: 4PO2) (Figure 1b) [15], which is the structure that resembles the corresponding complex structure of DnaK [22]. Although the αLid has no direct contact with the substrate, this lid is hypothesized to stabilize the SBD-substrate complex by sterically reducing the dissociation rate of the substrate [23]. The open αLid form in the domain-docked state significantly decreases substrate affinity with elevated association and dissociation rates of the substrate (Figure 1a), as demonstrated by the diminished affinity for the substrate in lidless DnaK [24]. 

DnaK lacking the αLid still changes substrate affinity in response to ATP binding to the NBD [25]. Experiments with DnaKs with a covalently fixed αLid helix A (αA) or B (αB) to βSBD demonstrated that detaching the αLid from the βSBD is not required for substrate binding, and the αLid opens to accommodate a folded part of the protein, suggesting that the αLid does not function as a lid to regulate the substrate binding ability of the SBD [26]. The role of the αLid in interdomain allostery for regulating substrate binding according to the nucleotide binding state of the NBD, therefore, seems rather limited despite the large structural change this lid undergoes in the chaperone cycle (Figure 1a). Instead, a conformational change to βSBD upon substrate binding likely plays pivotal roles in interdomain communication. 

Structural dynamics of βSBD in DnaK regulate its substrate affinity, with the conformation of βSBD rearranging between high-affinity and low-affinity states in a seesaw-like manner [27]. In the high-affinity state, interaction with a client-peptide causes the formation of a closed substrate binding cleft owing to the narrowing of the gap between L_1,2_ and L_3,4_ loops surrounding the cleft, which opens the other side of βSBD constituting the interdomain contact site. In the absence of the peptide, βSBD opens L_1,2_ and L_3,4_ loops with changes to the structure of the interdomain contact site [27]. In the domain-docked state of DnaK (ATP-bound form), four residues, K414, N415, Q442 and D481, located at the interdomain contact site of βSBD interact with residues in NBD to stabilize the interdomain contact [27]. The structural change in the interdomain contact site caused by substrate binding leads to rearrangement of these four residues, which diminishes the interdomain interaction leading to dissociation of the domains, which also releases the αLid from NBD. The released αLid covers the bound substrate in the cleft of βSBD to further stabilize the substrate bound form of SBD [27]. Disruption of the interdomain contact caused by substrate binding is supposed to conversely activate ATPase by releasing a subdomain in NBD, which explains the interdomain allostery from SBD to NBD [28]. 

As described above, the bacterial ortholog, DnaK, has been characterized extensively to explore the mechanism of interdomain communication, as a representative target in the Hsp70 family members that share the two-domain architecture. The sequence homology between DnaK and human Hsp70 (HSPA1A) is reasonably high (51% sequence identity in the full-length protein and 47% identity in the SBD), and thus similar overall folds of the SBDs are observed [15,22]. However, there are differences in the sequences between Hsp70 and DnaK for particular key residues (Figure S1). For example, K414, Q442 and D481 at the interdomain contact site of DnaK correspond to R416, E444 and N483 in Hsp70, respectively. In considering the allosteric mechanism for communication between the substrate binding site and the interdomain contact site within SBD, which relies on intimate interactions among the engaging residues, we hypothesize that these sequence differences will give rise to an alternative mechanism to achieve the intradomain and interdomain allostery in human Hsp70. By exploring this observation, we aim to enhance the significance of the recently reported substrate-bound structure of human Hsp70 SBD [15] as a molecular platform to design compounds that modulate the function of therapeutically crucial human Hsp70. 

With the above research aim, we determined the solution structure of human Hsp70 SBD, comprising βSBD and the short αLid with only the αA and αB helices, SBD(∆CDE) (Figure 1c). In SBD(∆CDE), the C-terminal half of αB is bound to the substrate binding cleft in an intramolecular manner, as previously found for similar constructs of other Hsp70 members [29,30,31,32]. A single amino acid change, L542Y, to SBD(∆CDE) causes the release of the αLid from the βSBD, as observed for DnaK [31]. The solution structure of the SBD with the L542Y mutation, SBD(∆CDE)-y, gives the first example of a human Hsp70 SBD structure in the apo-form. The SBD(∆CDE) structure represents the substrate-bound form of the βSBD subdomain, because of the structural independence between the C-terminal αB in the αLid and βSBD. Through structural comparison between SBD(∆CDE) and SBD(∆CDE)-y with additional analyses on the structural changes to SBD(∆CDE)-y upon client-peptide binding, we have explored the allosteric mechanism for stimulating the ATPase in the NBD following client-peptide binding to SBD. The present results demonstrate that the SBD in Hsp70 has a different way of allosteric communication within βSBD along with substrate binding when compared with that of the bacterial ortholog, DnaK. We also found that substrate binding to βSBD causes an allosteric change to disrupt the αLid-βSBD interaction to give rise to αLid conformational dynamics, which facilitates αLid contact with NBD to occur in the ATP-bound form of Hsp70. Taken together, the results describing the SBD of human Hsp70 provide a valuable comparison with data on the bacterial ortholog, DnaK, and revealed different and shared features of allosteric communication among residues in the SBD.

## 2. Results

### 2.1. Solution Structure of Human Hsp70 SBD(∆CDE)

**Figure 2:**
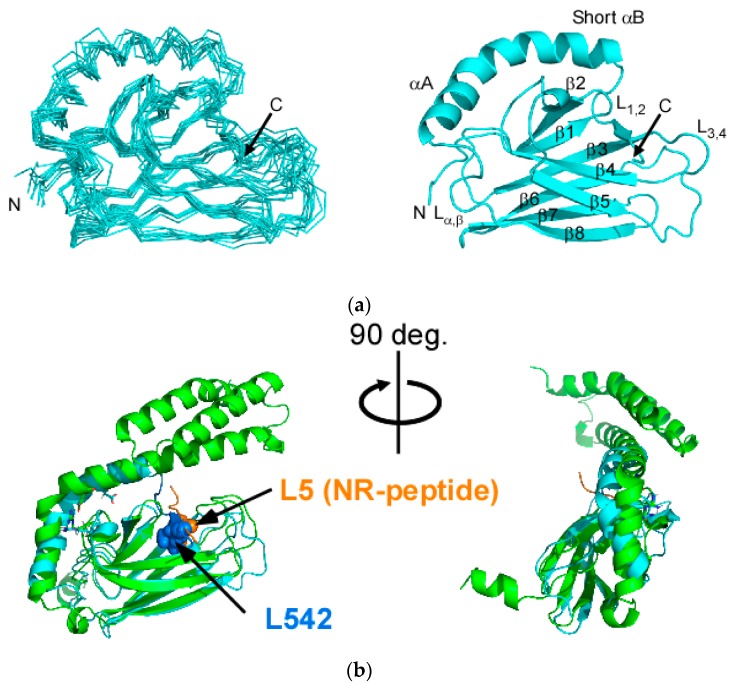
Solution structure of SBD(∆CDE) of human Hsp70. (**a**) Superposition of the 10 lowest-energy structures of SBD(∆CDE). The N- and C-termini are unstructured (residues 382–392 and 546–564) and were omitted for clarity (**left**). Ribbon representation of the lowest energy NMR structure of SBD(∆CDE) (**right**); (**b**) Superposition of the NMR structure of SBD(∆CDE) (cyan) and the crystal structure of the NR-peptide-bound SBD of Hsp70 (green) (PDB ID: 4PO2) [15]. Two different views are provided. Key residues in the substrate binding cleft of βSBD are drawn as spheres: L542 in SBD(∆CDE) (blue) and L5 in the NR-peptide with the sequence ^1^NRLLLTG^7^ (orange).

We solved the solution structure of SBD(∆CDE) comprising residues 382–564 (Figure 1c). As envisaged from other work on the SBD lacking αC, αD and αE (αCDE) in DnaK, Hsc70 and human Hsp70 (HSPA1A) [29,30,32,33], the C-terminal part of αB was unstructured and bound intramolecularly to the substrate binding cleft of the βSBD subdomain (Figure 2a). L542 in the A541-L542-E543 sequence of αB is buried in the binding cleft formed by L_1,2_ and L_3,4_ loops of SBD(∆CDE) (Figure 2a and Figure S2a). The binding mode of L542 to the cleft is similar to that found for L5 in the client-peptide of the sequence NRLLLTG (NR-peptide) bound to Hsp70 (Figure 2b) [15]. The structure of αB in SBD(∆CDE) is unstructured at V536 near the junction point residue N540 where the αB helix direction differs in molecules of the same asymmetric unit, showing the inherent structural flexibility of the αB subdomain [15]. The C-terminal part after the junction point in αB was also distinguishably labile from its N-terminal part in DnaK, as evidenced by the rapid hydrogen/deuteron exchange rates observed for the corresponding part of αB in the αLid [34]. 

The orientation of αA was slightly shifted from that found in the crystal structure of Hsp70-SBD with the NR-peptide (Figure 2b). The sideward movement of αA was also found in the solution structures of the SBD without αCDE in DnaK and Hsc70 [30,32]. The crystal structure of the Hsp70-SBD lacking αCDE having the C-terminal αB intramolecularly bound to the substrate binding cleft as found in SBD(∆CDE) (PDB ID: 4WV5) [29] retains the αA orientation as observed in the crystal structure of the peptide-bound Hsp70-SBD (PDB ID: 4PO2) [15]. In examining the crystal packing of the two molecules in an asymmetric unit of the crystal of the Hsp70-SBD lacking αCDE (PDB ID: 4WV5), the αA orientation in the crystal structure appears to be determined through the crystal contact [29]. The αA orientation in the solution structure of SBD(∆CDE) may represent the conformational change of αA induced by substrate binding (Figure 2b).

### 2.2. Solution Structure of the Hsp70 SBD that Lacks the Intramolecular αB Interaction, SBD(∆CDE)-y

**Figure 3:**
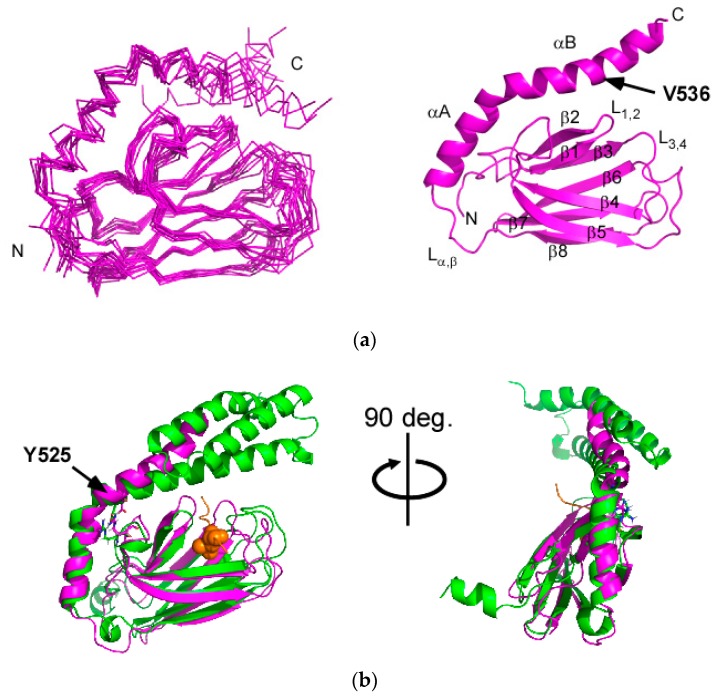
Solution structure of SBD(∆CDE)-y of human Hsp70. (**a**) Superposition of the 10 lowest-energy structures of SBD(∆CDE)-y with omission of the unstructured parts (residues 382–392 and 546–564) for clarity (**left**). Ribbon representation of the lowest-energy structure of SBD(∆CDE)-y (**right**); (**b**) Superposition of ribbon representations of the NMR structure of SBD(∆CDE)-y (magenta) and the crystal structure of the NR-peptide-bound form SBD in Hsp70 (PDB ID: 4PO2) (green). Two different views are provided. Residue L5 of the NR-peptide is shown by orange spheres [15].

Changing L542 to tyrosine in SBD(∆CDE) eliminated intramolecular binding of the C-terminal part of αB in the αLid to the substrate binding cleft in βSBD, which enabled determination of the SBD structure in the apo-form, which has not been solved previously for Hsp70 (Figure 3a). In the following discussion, the mutant SBD is referred to as SBD(∆CDE)-y. 

DnaK SBD (residues 387–552) with the related mutations (L542Y/L543E) forms a stable apo-form of the isolated SBD [31]. DnaK with the mutant SBD (L542Y/L543E) recovers substrate-binding activity and shows substrate-induced ATPase activation [31]. DnaK with the SBD with the intramolecular interaction to the C-terminal part of αB does not respond to the substrate-peptide but maintains a higher ATPase activity, which is presumably because the SBD persistently stays in a substrate-bound form because of intramolecular αB binding to the substrate binding cleft [31]. The elevated ATPase activity found for the DnaK with the SBD in an intramolecular-bound form suggests that the self-binding form of SBD mimics the client-peptide bound form of βSBD and the αA part in the αLid. 

The solution structure of SBD(∆CDE)-y shows that the αB in the αLid is released from the binding cleft in βSBD (Figure 3a). L542 in SBD(∆CDE) must be spatially close to T429 because of the observed NOE, whereas the corresponding NOE was absent in SBD(∆CDE)-y (Figure S2). The αB helix in SBD(∆CDE)-y was extended and consisted of residues 526–543 (Figure 1c). The extended helix part of αB in SBD(∆CDE)-y is still structurally flexible, as indicated by the lower ^1^H-^15^N heteronuclear NOE (hNOE) values (Figure S3). This flexibility is ascribed to the lack of the helix bundle structure with αCDE and the inherent structural flexibility of the C-terminal part of αB, as demonstrated in DnaK (Figure 1c) [31]. The αB is kinked at the junction position V536, which is the starting point of the extended part of αB. The helical property change in αB at the junction coincides with that observed between two crystal forms of the substrate-bound SBD of Hsp70 in an asymmetric crystal unit [15]. 

The αA direction in the substrate free form of SBD(∆CDE)-y is close to that found in the crystal structure of the substrate-bound form of SBD in Hsp70 (Figure 3b). αB rotates upward at the hinge residue Y525 that connects αA and αB, and this differs when compared with the corresponding αLid part of the substrate-bound Hsp70 SBD crystal structure (Figure 3b). The αLid in the apo-form does not cover the substrate binding cleft but remains open to promote client protein binding to the cleft, which is consistent with the conformational equilibrium of the αLid observed in the ADP-bound state of DnaK [23].

### 2.3. Structural Comparison of SBD(∆CDE) and SBD(∆CDE)-y

**Figure 4:**
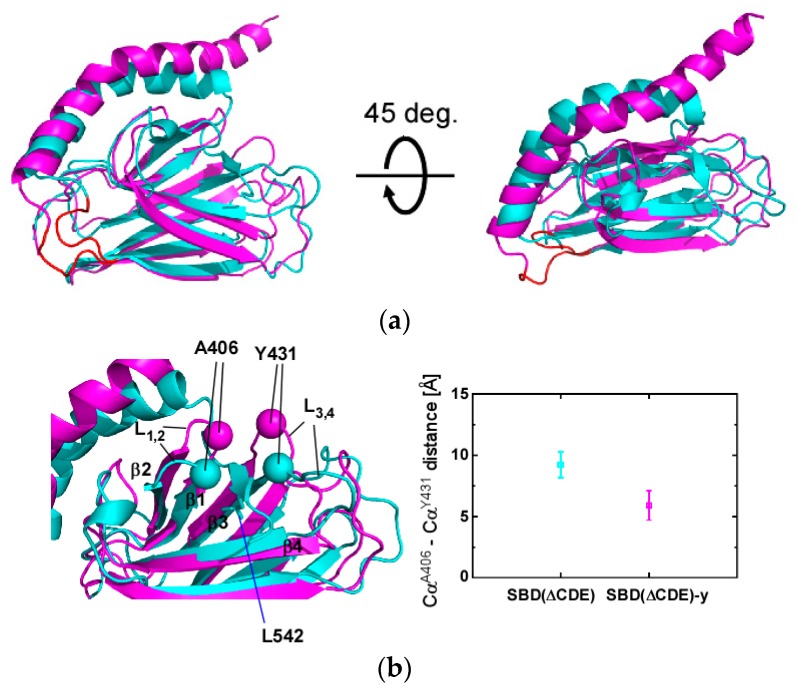

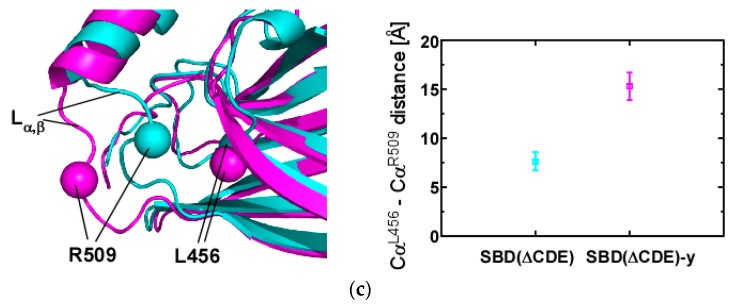
Structural comparison between SBD(∆CDE) and SBD(∆CDE)-y of human Hsp70. (**a**) Superposition of the lowest-energy NMR structures of SBD(∆CDE) (cyan) and SBD(∆CDE)-y (magenta). The L_α,β_ loop in both structures is marked in red; (**b**) Comparison of the substrate binding loops L_1,2_ and L_3,4_. Cα atoms in residues at the tip of the loops are show as spheres: A406 (L_1,2_) and Y431 (L_3,4_) (**left**). The average and standard deviation of the A406–Y431 Cα distance for the 10 lowest-energy structures of SBD(∆CDE) (cyan) and SBD(∆CDE)-y (magenta) are presented (**right**); (**c**) Structural comparison of the L_α,β_ loop between SBD(∆CDE) (cyan) and SBD(∆CDE)-y (magenta). Cα atoms of the residue located in the middle of the L_α,β_ loop (R509) and L456 in β5 are shown as spheres (**left**). The average and standard deviation of the L456-R509 Cα distance for the 10 lowest-energy structures of SBD(∆CDE) (cyan) and SBD(∆CDE)-y (magenta) are presented (**right**).

Residues 382–535 of the SBD(∆CDE) (prior to the junction position for the αB structure) and SBD(∆CDE)-y are supposed to represent the SBD in the substrate-bound form and apo-form, respectively (Figure 4a). This is supported by observations for DnaK with the SBD lacking αCDE, which retains the ability for the interdomain allosteric communication as the full-length protein. Here, DnaK with the self-binding SBD shows a similar interdomain allosteric effect to activate the ATPase, although it does not bind to an auxiliary client-peptide, whereas DnaK with the mutant SBD releasing αB from the binding cleft elevates the ATPase activity in response to client-peptide binding [31]. 

The structure of the L_1,2_ and L_3,4_ loops changed upon binding to the C-terminal segment of αB comprising A541-L542-E543 as an intramolecular substrate in SBD(∆CDE) (Figure 4b). The binding of the segment causes the distance between the loops to widen with the distance between the Cα atoms of A406 and Y431 (Figure 4b) changing from 5.9 ± 1.2 Å in the apo-form SBD(∆CDE)-y to 9.2 ± 1.0 Å in the substrate-bound form SBD(∆CDE) (Figure 4b). The corresponding Cα distance in the NR-peptide-bound crystal structure of Hsp70-SBD was 7.1 Å (Figure S4). The inter-loop structure should change to adopt a structure similar to that found in the substrate bound state.

Conversely, substrate binding to βSBD in DnaK led to the corresponding residues M404 (L_1,2_) and A429 (L_3,4_) to move towards each other [27]. Thus, the conformational change in the substrate binding loops differs between Hsp70 and DnaK. 

The other notable structural change was found in the L_α,β_ loop that connects βSBD to the αLid (Figure 4c). Here, the Cα distance between R509 and L456 changed from 15.3 ± 1.4 Å in the apo-form to 7.6 ± 0.9 Å in the substrate-bound form (Figure 4c). The hNOE values for residues of the L_α,β_ clearly indicate that this loop is intrinsically flexible (Figure S3), but the NMR structural ensemble of the L_α,β_ loop in the apo- and the substrate-bound states are different to each other, which suggests the structure and dynamics of the L_α,β_ loop should change according to substrate binding; as representative spectral changes, the NOEs observed between L456 and R509 in SBD(∆CDE) were absent in SBD(∆CDE)-y (Figure S5). 

The intramolecular interaction of the C-terminal part of αB to the substrate binding cleft caused significant chemical shift changes at the edges of the L_α,β_ loop (Figure S6), which might be related to the conformational change to flip L_α,β_ from the position in the apo-form SBD(∆CDE)-y to that in the substrate-bound form SBD(∆CDE) (Figure 4c). The chemical shift changes suggest an allosteric connection between residues in the substrate binding cleft and the L_α,β_ loop in the interdomain contact site; similar allostery among residues within the SBD of DnaK has also been observed [27,28].

### 2.4. Substrate Binding Causes Chemical Shift Changes at the Hinge Between αA and αB in the αLid

**Figure 5:**
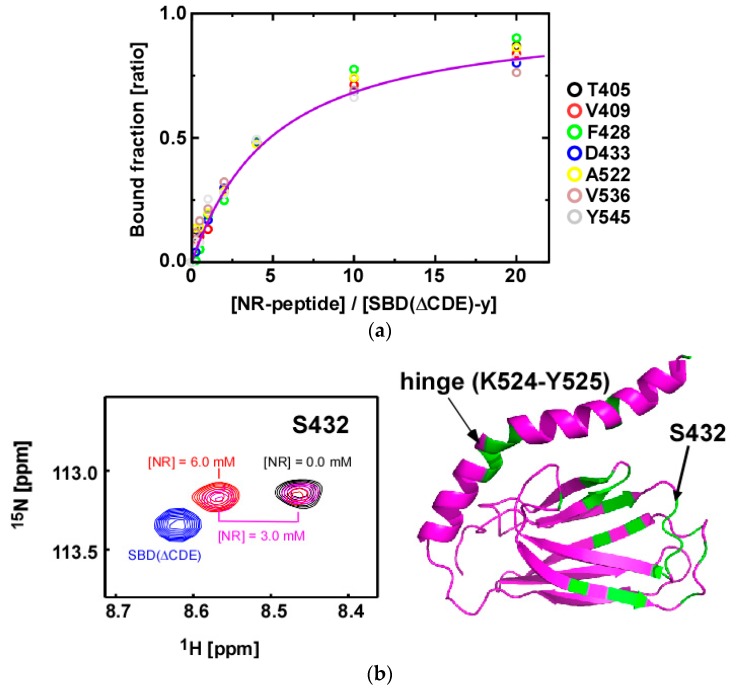
NR-peptide binding to SBD(∆CDE)-y of human Hsp70. (**a**) Determination of the binding affinity of the NR-peptide to SBD(∆CDE)-y by measuring NMR signal intensity changes in 2D ^1^H-^15^N HSQC spectra for the listed residues examined in the NR-peptide titration. Global fitting the bound fractions of the listed residues gave a *K*_D_ = 1.3 ± 0.1 mM; (**b**) A representative change in resonance intensities observed during the NR-peptide titration with SBD(∆CDE)-y. Data for S432 is shown (**left**). When the NR-peptide concentration ([NR]) is 3.0 mM and 0.3 mM SBD(∆CDE)-y is present there are signals observed for both the bound and free states (magenta). At [NR] = 6.0 mM, only the bound state signal for S432 is observed (red). For comparison, the 2D ^1^H-^15^N HSQC signal for S432 in SBD(∆CDE) (intramolecular αB bound form) is shown in blue. Residues showing significant spectral changes observed at [NR] = 1.2 mM are mapped onto the solution structure of SBD(∆CDE)-y (**right**).

We examined the substrate binding affinity of SBD(∆CDE)-y to the NR-peptide by NMR (Figure 5a). The NMR titration experiments demonstrated that the substrate binding process is in the slow exchange regime on the chemical shift time scale, giving discrete bound-state signals upon addition of the peptide (Figure 5b). The *K*_D_ value was determined by measuring changes in the signal intensity relative to that in the free-state SBD(∆CDE)-y (Figure 5a) to yield a *K*_D_ = 1.3 ± 0.1 mM. The isothermal titration calorimetry (ITC) experiments for the NR-peptide binding to SBD(∆CDE)-y corroborated the NMR results with a *K*_D_ = 1.5 ± 0.3 mM (Figure S7). The observed binding affinity of SBD(∆CDE)-y to the NR-peptide is similar to that found for the low-affinity state of Hsp70 in the ATP-bound state, i.e., *K*_D_ = 1.25 ± 0.02 mM [10]. The results for SBD(∆CDE)-y do, however, contrast those for the related SBD construct of DnaK (387–552) with L542Y and L543E mutations abrogating intramolecular αB binding to βSBD. DnaK SBD with the double mutation L542Y/L543E retains high affinity toward client peptides, as observed for the wild-type full-length DnaK in the high-affinity state (ADP-bound state) [31]. The discrepancy may be due to the different modes of client peptide recognition between Hsp70 and DnaK, as illustrated in the crystal structures [15,22]. 

The observed spectral changes to data recorded on SBD(∆CDE)-y upon peptide binding demonstrates slow binding kinetics with respect to the average chemical shift difference of 58 Hz in the ^1^H dimension, irrespective of its low affinity (Figure 5b). From a simulation using the average *R*_2_ rate of 21 s^−1^ for residues engaged in peptide binding and *K*_D_ = 1.3 mM, we estimated that the maximum *k*_off_ will be 30 s^−1^ to fulfill the condition that the chemical shift changes for the free-state SBD(∆CDE)-y upon binding are limited to less than 5 Hz along the ^1^H dimension, as observed for the SBD(∆CDE)-y (0.3 mM) binding to the peptide (3.0 mM) (Figure 5b) [35]. This gives a *k*_on_ = 2.3 × 10^4^ M^−1^ s^−1^, which is consistent with the kinetics for the lidless SBD of DnaK in binding to its target peptide (*k*_on_ = 2 × 10^4^ M^−1^ s^−1^ and *k*_off_ = 12.5 s^−1^) [25]. 

The reduction in the binding ability of SBD(∆CDE)-y when compared to that of the SBD in the full-length Hsp70 in the ADP-bound form is ascribed to the unstable structure of the C-terminal part (after the junction residue V536, Figure 3a), whose structural instability is indicated by the lower hNOE values (Figure S3d). In DnaK, αLid is shown to play a role to extend the life time of the substrate-SBD complex by sterically blocking the substrate release from the binding cleft in βSBD [24]. The structurally flexible C-terminal αB in αLid due to the lack of αCDE helix bundle impairs the function to stabilize the substrate bound complex form. 

αCDE helix bundle stabilizes the C-terminal part of the αB structure through the physical contact as observed in the crystal structure of the Hsp70-SBD (Figure 1b) [15]. The C-terminal part of αB is structurally labile even in the presence of αCDE helix bundle, as evidenced by the rapid H/D exchange to the amide proton of the residues in the corresponding part in DnaK [34]. The C-terminal αB frequently becomes unstructured by opening the hydrogen bonds. αCDE presumably keeps the αB part unstructured transiently. The lack of αCDE, therefore, populates the unstructured αB having leucine as a preferential binding residue to be recognized as a client by βSBD, which is observed in the solution structure of SBD(∆CDE) (Figure 2a). 

The NR-peptide binding to SBD(∆CDE)-y caused significant spectral changes to the hinge between αA and αB in the αLid (Figure 5b), which suggests an induced conformational change to the αLid upon binding of the peptide to βSBD. In this weak client peptide binding, the spectral change to L_α,β_ was less significant (Figure 5b), which contrasts that observed for SBD(∆CDE) with intramolecular αB binding (Figure S6). 

**Figure 6:**
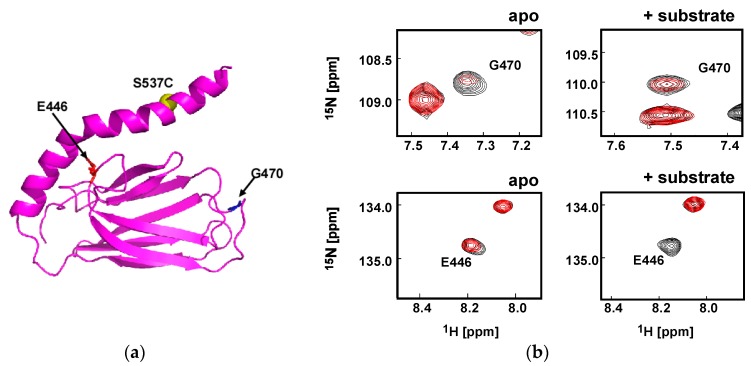
PRE experiments with SBD(∆CDE)-y with spin labeling at residue 537. (**a**) The spin-labeling position in the solution structure of SBD(∆CDE)-y is shown, in which, S537 was exchanged to cysteine to facilitate covalent labeling with MTSL; (**b**) Representative spectral changes caused by the paramagnetic effect. In the left two panels, 2D ^1^H-^15^N HSQC spectra are overlaid for the paramagnetic spin labeled sample (red) and for the sample with a diamagnetic label (black) in the absence of the NR-peptide. In the two panels on the right, the corresponding spectral comparison in the presence of 5.0 mM NR-peptide. The concentration of SBD(∆CDE)-y was adjusted to 0.5 mM.

To further characterize the αLid structural change upon binding of the NR-peptide to SBD, we used paramagnetic relaxation enhancement (PRE) experiments with the SBD(∆CDE)-y harboring a S537C mutation to enable nitroxide labeling (Figure 6a). In the PRE experiments, we compared the signal intensities of each cross peak in the 2D ^1^H-^15^N HSQC spectra collected for the sample labeled with the paramagnetic nitroxide radical and the spectrum recorded for the protein with the diamagnetic label to elucidate the spatial proximities of the residues to the nitroxide radical at residue 537. Reduction in signal intensities indicates that the corresponding residue associated with that signal are spatially proximate to the radical (within approximately 25 Å), and for shorter distances less than about 10 Å, the spin-label bleaches the signals completely [36]. Two representative signals are provided in Figure 6b to demonstrate the change in the structure of the αLid upon binding the NR-peptide. In the apo-form, the spin-label at 537 isclose to G470 in the L_5,6_ loop, whereas this residue moved away from the spin-label upon NR-peptide binding (Figure 6b). Residue E446 spatially neighboring the hinge between αA and αB in the apo-form SBD(∆CDE)-y structure becomes closer to the spin-label upon peptide binding (Figure 6b). 

The distance of the Cα atoms between E446 and S537 in the lowest energy SBD(∆CDE)-y NMR structure is 27 Å, whereas the Cα distance between G470 and S537 is 26 Å (Figure 6b). The PRE data suggest that αB is in conformational equilibrium to allow the spin-label to move closer to G470, which is consistent with the αLid conformational dynamics observation in DnaK [23,26]. The enhanced PRE effect on E446 (Figure 6b) and the spectral changes to residues at the hinge between αA and αB in the αLid (Figure 5b) upon NR-peptide binding suggest that the αLid becomes kinked at the hinge, which moves the spin-label closer to E446. 

The change in the PRE effect for residues in SBD(∆CDE)-y upon peptide binding further support the view that the αLid becomes more dynamic to affect a wider range of residues in SBD(∆CDE)-y upon binding of the NR-peptide (Figure S8). As found for the SBD in DnaK, the αLid subdomain in SBD of Hsp70 is not always positioned over the substrate binding cleft but becomes more dynamic when the peptide is bound than that observed in the apo-form [23,26].

## 3. Discussion

### 3.1. Client-Peptide Binding Changes the Structure at the Lynchpin Site for the Interdomain Contact

The present work provides structural insights into the interdomain allosteric communication from SBD to NBD in human Hsp70 by structural comparison of SBD(∆CDE) and SBD(∆CDE)-y: SBD(∆CDE) represents the substrate-bound form of SBD (Figure 2a), whereas SBD(∆CDE)-y represents the apo-form SBD structure (Figure 3a). SBD(∆CDE) showed intramolecular interactions of the C-terminal part of αB in the αLid (Figure 2a). In considering the distinct structural flexibility of the C-terminal αB observed in DnaK [31], we postulate that the βSBD and residues up to the junction position V536 in the αLid, at which point the structural stability of αB alters, could represent the substrate bound form of SBD (Figure 4a). Because binding of the NR-peptide to SBD(∆CDE)-y was too weak to obtain a stable substrate-bound form of SBD (Figure 5), the solution structure of SBD(∆CDE) is more reliable to explore the structural changes that likely happen in βSBD and the N-terminal part of the αLid upon substrate binding to the binding cleft in the SBD. 

DnaK harboring the SBD but lacking the entire part of the αLid retains interdomain allosteric communication [25], suggesting that the αLid conformational switch does not have a primary role in achieving allosteric interdomain communication (Figure 1a). Instead, recent work on DnaK stresses the functional significance of the conformational change in βSBD upon binding the client-peptide [27,28]; structural rearrangement occurs to residues engaged in the interdomain interaction between βSBD and NBD. 

**Figure 7:**
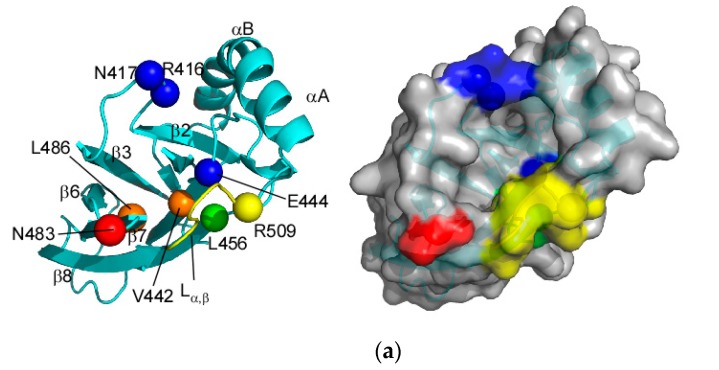

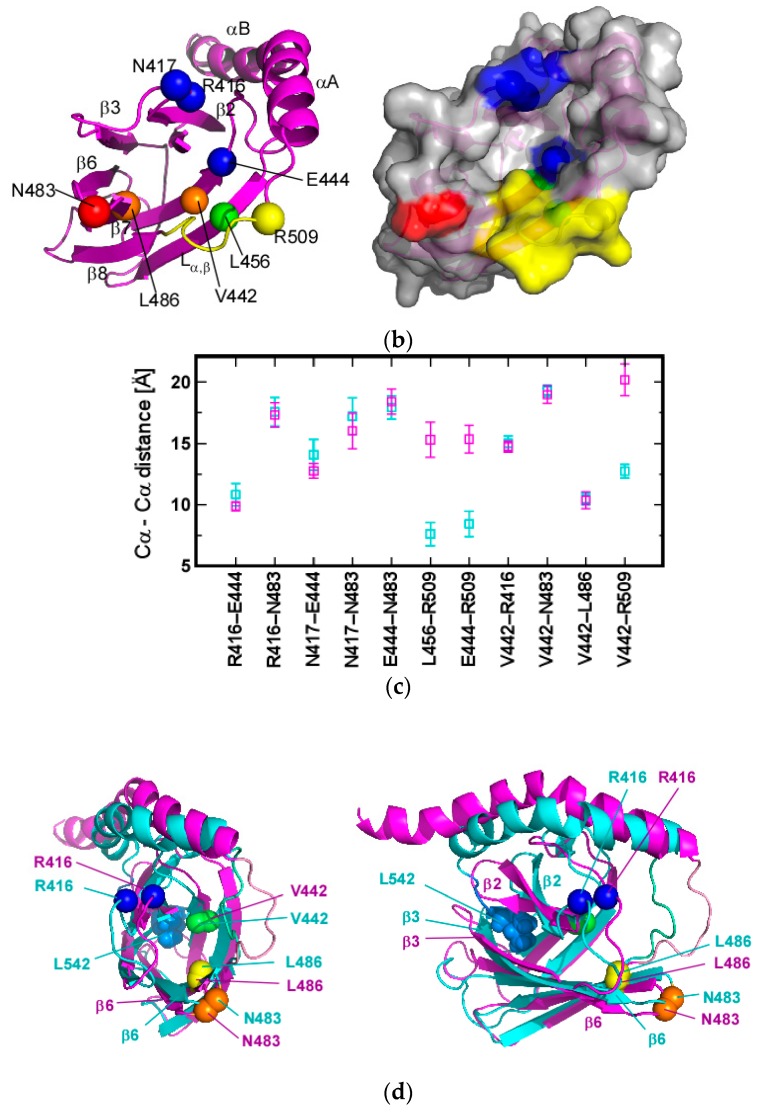
Structural changes to residues located at the interdomain interface. (**a**) The Cα positions of residues engaged in the interaction with NBD: the Cα atoms for the annotated residues are shown by spheres on the structure of SBD(∆CDE) (**left**). A surface plot of the structure is shown in the left panel (**right**); (**b**) The corresponding residues marked in SBD(∆CDE) are shown in the structure of SBD(∆CDE)-y (**left**) with its surface plot (**right**); (**c**) Cα distance distributions for pairs of residues among the NMR ensemble of SBD(∆CDE) (cyan) and SBD(∆CDE)-y (magenta); (**d**) Structural comparison between SBD(∆CDE) (cyan) and SBD(∆CDE)-y (magenta) demonstrates the Cα position changes for pivotal residues engaged in interdomain interactions.

The structural comparison between SBD(∆CDE) (substrate-bound form) and SBD(∆CDE)-y (apo-form) revealed two notable conformational changes in the interdomain interface site in SBD of Hsp70 (Figure 7a,b). One conformational change occurs in L_α,β_, and the other results from the displacement of R416, N417 and N483 (Figure 7c). 

The two domains within DnaK in the ADP bound state behave independently and are detached from each other [17]. In the ADP bound form of DnaK, the client-peptide-bound SBD shows that the L_α,β_ loop masks Q442, which presumably hampers interdomain contact between Q442 and D148 and thus ensures that the domains remain separated (PDB ID: 2KHO) [17,27]. 

The crystal structure of DnaK in the ATP-bound form (PDB ID: 4B9Q) showed that residues K414, N415, Q442 and D481 in the SBD interact with residues in the NBD. The pairs of interacting residues are as follows: (K414 and N415)–D326, Q442–D148 and D481–I168 [18]. The DnaK D481N mutant was shown by NMR to have a reduced contact frequency of the βSBD with the NBD in the ATP bound state [21], indicating that D481 directly connects the two domains in solution. Mutations to residues K414 and D481 enhanced the basal ATPase activity through impairing their interactions to residues in the NBD, which shows that the interdomain contact causes the NBD to adopt a conformation that is incompetent for ATP hydrolysis [28]. DnaK with the K414I mutation maintains client-peptide binding ability, but this mutation abrogates the two-way interdomain allostery, client-peptide stimulation to ATPase activity and the ATP induced peptide release, which demonstrates the pivotal role of K414 in the functional linkage between the domains [37].

The allosteric coupling between the substrate binding site and the interdomain contact site within the SBD was observed in DnaK [27,28], which explains the substrate-binding induced elevation of ATPase activity. Kytik and coworkers proposed a mechanical model for the intradomain allostery, in which the substrate in the cleft pushes residues V440 and L484 away to subsequently dislocate D481 and thus impair the D481-I168 interdomain interaction [28]. Zhuravleva and Gierasch argued that substrate binding prohibits the seesaw-like conformational dynamics of the SBD, thus giving rise to a fixed structure with closed substrate binding loops, L_1,2_ and L_3,4_, with simultaneous rearrangement of residues K414, N415, Q442 and D481 to positions that can no longer engage in interdomain contacts [27]. Zhuravleva and Gierasch also noted that client-peptide binding altered the conformation of the L_α,β_ loop to inhibit Q442 binding to NBD [27]. 

The corresponding residues to the above discussed residues in DnaK are marked in the structures of SBD(∆CDE) and SBD(∆CDE)-y (Figure 7a,b). Residues R416, N417 and N483 in Hsp70 are K414, N415 and D481 in DnaK. The change in the inter-atomic Cα distances for residues at the interdomain contact site between SBD(∆CDE) (bound form) and SBD(∆CDE)-y (apo-form) indicate that these residues are displaced by substrate binding (Figure 7c). Although the Cα distance between R416 and N483 does not show a large change upon substrate binding (Figure 7c), the spatial arrangement of the residues in the interdomain contact site is clearly visible (Figure 7d). Disarrangement of R416 and N483 may weaken the interdomain contact and subsequently promote ATP hydrolysis, as demonstrated for DnaK [28]. Residues V442 and L486 move to dislocate N483 upon peptide binding, as similarly observed for V440 and L484 in DnaK [28]. 

The dislocation of R416 upon peptide binding is caused by the rearrangement of the β2 in adopting the client-peptide (Figure 7d). Peptide binding to the cleft widens the distance between the L_1,2_ and L_3,4_ loops, which is opposite to the conformational change observed for DnaK (Figure 4b). In DnaK, the substrate binding loops are closer to each other upon peptide binding to the cleft [27]. The conformational change to L_1,2_ upon substrate peptide binding deformed the β2 to displace R416 (Figure 7d). The β2 structure in SBD(∆CDE) is close to that in the crystal structure of the peptide-bound form of the Hsp70-SBD [15]. The extent of the β2 structural change appears to correlate with the degree of R416 dislocation, as seen by comparison of the crystal structures of Hsp70 SBD and SBD(∆CDE) (Figure S12b). The structural comparison also shows that the position of R416 changes according to the bound substrate sequence, which suggests that the interdomain contact is modulated according to the bound substrate.

E444 in Hsp70 is accessible by NBD in the apo-form SBD(∆CDE)-y, whereas it is covered by the L_α,β_ loop and therefore this interaction is hindered in the substrate-bound form (Figure 7a,b). This is consistent with the observation for Q442 in DnaK, as described above [27]. The role of E444 in the interdomain interaction was confirmed by the crystal structure of the SBD in Hsp70 with covalently modified E444 by novolactone (PDB ID: 4WV7); the chemical modification of E444 by novolactone sterically blocks the interdomain interactions [29]. 

The Hsp70 SBD modified by novolactone contains βSBD as well as αA and αB in the αLid (residues 395–543) [29]. The crystal structures of the SBD both in the apo-state and novolactone bound forms showintramolecular binding of a part of αB with L542 being buried in the hydrophobic cleft (PDB ID: 4WV5, and 4WV7) [29], which is consistent with the solution structure of SBD(∆CDE). In both crystal structures, the L_α,β_ loop conformation was not determined because of its intrinsic structural flexibility [29]. In the crystal structure of the Hsp70-SBD bound with the NR-peptide, one of the two molecules in the asymmetric unit gives the L_α,β_ structure stabilized by crystal packing through interactions with a loop from an adjacent molecule, whereas the other molecule does not show the loop structure (PDB ID: 4PO2) [15]. The structures of SBD(∆CDE) and SBD(∆CDE)-y in this work are, therefore, valuable because they provide the structures of the L_α,β_ loop (Figure 4c). 

L456 in Hsp70 as a counterpart residue to L454 in DnaK is also covered by L_α,β_ in the peptide-bound form SBD(∆CDE) (Figure 7a,b), which may impair the interdomain contact, as envisaged from the observation that DnaK with the L454I mutation shifted the conformational equilibrium to the domain-undocked state [27]. 

**Figure 8:**
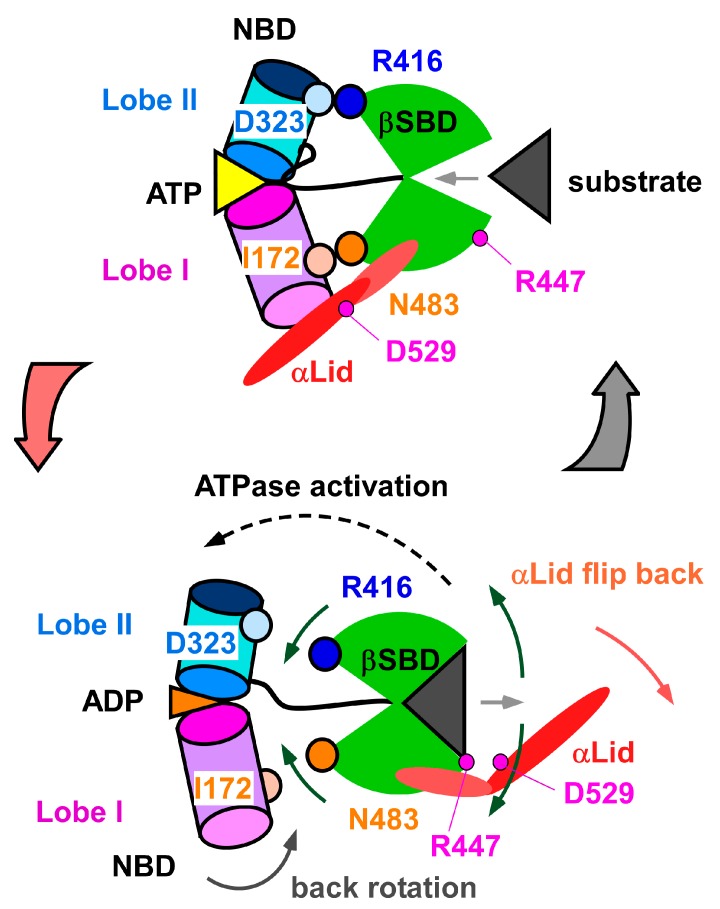
Schematic showing interdomain communication with structural changes to both the SBD and NBD in association with client-peptide binding, and ATP hydrolysis and ADP turn over. The client-peptide (substrate) binding to SBD causes rearrangement of residues at the interdomain contact site to impair the interaction between SBD and NBD, which induces the back rotation of Lobe I in NBD to activate ATP hydrolysis. Substrate binding should also disrupt the inter-subdomain interaction that connects the αLid to the βSBD and is mediated by a salt bridge between R447 and D529. Such disruption of this interaction releases the αLid from βSBD and promotes the flip movement of the αLid to the NBD. The elevated αLid motion by disrupting the R447–D529 interaction also facilitates substrate release to promote progression of the chaperone cycle. The structural change in the lynchpin site for the inter-domain contact and also the intradomain allosteric structural change within SBD upon substrate binding are intimately related to peptide and nucleotide turnover, and their inter-relation between the structural change and turnover process promotes progression of the chaperone cycle of Hsp70.

Taken together, the conformational changes at the interdomain interface that dislocate or conceal residues engaged in contact to the NBD upon binding the client-peptide will shift the population to the domain-undocked state and subsequently elevate ATPase activity to promote progression of the chaperone cycle (Figure 8). According to the proposed mechanism for the interdomain communication in DnaK [28], the domain-undocked state of Hsp70 will become populated by disruption of the interaction between N483 and I172 (D481 and I168 in DnaK), which allows the back rotation of Lobe I in the NBD to promote ATP hydrolysis (Figure 8). Once the SBD is released from contact with the NBD upon peptide binding, the L_α,β_ loop conformation changes to mask E444 and L456 from their contact to NBD, which will further stabilize the domain-undocked form of Hsp70 (Figure 7a,b).

### 3.2. Change in αA Orientation upon Substrate Binding May Pave the Other Way for Interdomain Communication between SBD and NBD

In the peptide-bound form, SBD(∆CDE), αA adopted a different orientation when compared with that of the apo-form, SBD(∆CDE)-y (Figure 7). The change in the orientation of αA was also observed in the solution structures of SBD in DnaK and Hsc70. The intramolecular binding of the C-terminal part of the αB in the αLid of these two proteins is similar to that observed in SBD(∆CDE) [30,32]. Although a functional role for the disorientation of αA was not described for DnaK and Hsc70, we hypothesize that reorientation of αA upon peptide binding has a functional role. 

The salt bridge between the side chain of R447 and D529 in the crystal structure of Hsp70 SBD complexed with the NR-peptide [15] corresponds to the interaction observed for residues R445 and D526 in DnaK [22,38]. The loss of the interaction between R447 and D529 in SBD(∆CDE) was confirmed by changes in the NOE pattern, which provides structural restraints that connect the αLid to βSBD (Figure S10). Residues R447 and D529 in SBD(∆CDE) do not interact with each other, whereas the two residues in SBD(∆CDE)-y are located close to the respective residues in the crystal structure of Hsp70 SBD, suggesting that these residues in SBD(∆CDE)-y could form a salt bridge (Figure S11). 

The αLid conformation in the crystal structure of the Hsp70-SBD substrate-bound form is possibly determined by an intermolecular contact between two molecules in a crystal unit (PDB ID: 4PO2) [15]. The loss of the salt bridge between R447 and D529 in the solution structure of the substrate-bound SBD(∆CDE) suggests that substrate binding destabilizes the interaction between the αLid and βSBD. 

Chemical shift changes for resonances associated with residues at the hinge between αA and αB were observed following NR-peptide binding to SBD(∆CDE)-y (Figure 5b). This observation suggests that peptide binding changes the hinge structure near D529 in the αLid (Figure S11). The substrate induced structural change to the hinge may explain why substrate binding disrupts the neighboring salt bridge interaction between R447 and D529. Besides the chemical shift changes for resonances corresponding to residues at the hinge in the αLid, PRE experiments showed that peptide binding facilitated movement of the αLid away from the βSBD, which enabled the spin-label at residue 537 to move closer to E446 and the hinge in the αLid (Figure 6 and Figure S8). The results demonstrate that the αLid does not stay in the substrate-binding site to cover the bound peptide, as found in the peptide-bound Hsp70-SBD crystal structure (Figure 1b). Instead, the αLid becomes more dynamic upon peptide binding in solution, presumably through disruption of the R447–D529 salt bridge that connects the αLid to βSBD. 

Impairing the inter-subdomain contact between the αLid and βSBD mediated by the R445–D526 interaction was shown to enhance the kinetic process of client-peptide binding to the substrate binding site in DnaK [26,38]; the peptide association and dissociation rates increased by ~160-fold and ~27.4-fold, respectively [38]. It was also demonstrated that the αLid does not always function as a lid to protect client-peptide release in the substrate-bound state of DnaK; the αLid can be flexible when bound to a globular protein but not to an unstructured peptide, and this arises from the rupture of the interaction between R445 and D526 [26]. In the ATP-bound DnaK, αA in the αLid flips to bind to the Lobe I in the NBD (Figure 1a) [18], in which state the R445–D526 interaction is absent [26]. The R445–D526 interaction, therefore, has a role in switching the αLid function. In particular, breaking the R445–D526 interaction should occur prior to the chaperone moving back into the ATP-bound state to enable αLid reorientation from the SBD to Lobe I in the NBD (Figure 1a). 

Breaking the salt bridge between R447 and D529 upon peptide binding in SBD(∆CDE) (Figure S11) should promote formation of an open lid conformation (Figure 1a). There has been no hypothesis for substrate binding to promote the αLid to flip to the NBD, because the crystal structures of the peptide bound form of the SBD in Hsp70 and DnaK retain the salt bridge to fix the αLid to βSBD [15,22]. The present results from the solution structure of SBD(∆CDE) and the conformational changes observed for SBD(∆CDE)-y in binding to the client-peptide provide a possible mechanism where substrate binding enhances αLid motion by breaking the salt bridge between R447 and D529. We postulate that the probability of rupture of the R447–D529 interaction should depend on the properties of the bound substrate, which includes sequence, structure and affinity, because the structural change in βSBD seems tunable according to the interaction with the bound peptide as found in the structural comparisons presented herein (Figure S12). This implies that the bound substrate itself alters the progression of the chaperone cycle of Hsp70. 

We should additionally note the disruption of the R447–D529 interaction is more plausible in Hsp70 than in DnaK. In the corresponding interaction in DnaK, D526 forms ionic interactions to two residues R445 and K446 in βSBD, whereas D529 interacts only with R447 in Hsp70; the corresponding residue of K446 in DnaK is A448 in Hsp70. We cannot completely rule out the possibility that the rupture of the R447–D529 interaction is caused by distortion of the αLid caused by the intramolecular interaction via the C-terminal part of αB in SBD(∆CDE). However, PRE and spectral intensity changes caused upon substrate binding support the proposed mechanism for peptide binding disrupting the αLid-βSBD interaction to switch the functional mode of the αLid.

## 4. Materials and Methods 

### 4.1. Sample Preparation

The gene of human Hsp70 SBD (UniProt accession No P08107) was cloned into pET16b using NdeI and XhoI sites, and transformed into *E. coli* BL21(DE3). All site-directed mutagenesis to the SBD were conducted by KOD FX NEO (Toyobo, Osaka, Japan). For NMR sample preparation, cells were grown in M9 minimal medium with 100 μg/mL ampicillin at 37 °C to an OD600 = 0.6–0.8. ^15^NH_4_Cl and ^13^C_6_-glucose were used as nitrogen and carbon sources, respectively. Protein overexpression was induced by isopropyl β-d-thiogalactopyranoside (IPTG, final conc.: 1.0 mM), followed by an additional 4 h incubation at 37 °C. Cells were harvested by centrifugation and resuspended in buffer A (50 mM Tris-HCl (pH 8.0), 150 mM KCl) and then subjected to sonication. Cell debris was removed by centrifugation and the supernatant was loaded onto a HisTrap FF column (GE Healthcare, Waukesha, WI, USA) equilibrated with buffer A. The column was washed with buffer B (50 mM Tris-HCl (pH 8.0), 150 mM KCl, 20 mM imidazole) and the target protein eluted by buffer C (50 mM Tris-HCl (pH 8.0), 150 mM KCl, 500 mM imidazole). The N-terminal His_6_-tag was cleaved by Factor Xa (250 units; Novagen, Madison, WI, USA) during dialysis against buffer D (50 mM Tris-HCl (pH8.0), 10 mM KCl) at 23 °C for 16 h. The tag-cleaved SBD was purified using a HiTrap Q FF column (GE Healthcare) with a KCl gradient (from 0.01 to 1 M) in buffer D. The proteins were dialyzed at 4 °C for 12 h against 3 changes of buffer E (50 mM potassium phosphate (pH 7.0), 50 mM KCl). For ITC samples, cells were grown in LB broth at 37 °C. Protein overexpression was induced by adding IPTG to a final concentration of 1 mM and cells were cultured for a further 18 h at 16 °C. The protein was purified by the same procedure described above.

### 4.2. NMR Spectroscopy and Structure Determination

Structures of SBD(∆CDE) and SBD(∆CDE)-y were determined according to a standard procedure using uniformly ^13^C/^15^N labeled samples [39]. A set of standard three-dimensional (3D) triple-resonance data was used to assign backbone and side chain resonances. NOEs from 3D ^15^N-edited NOESY (mixing time: 80 ms) and 3D ^13^C-edited NOESY (mixing time: 80 ms) spectra provided distance restraints used in the structure calculations. All the NMR data were collected on an Avance II spectrometer (Bruker BioSpin, Ettlingen, Germany) equipped with a triple-resonance cryogenic probe operating at a ^1^H resonance frequency of 700 MHz. The protein sample was dissolved in a buffer solution consisting 50 mM potassium phosphate (pH 7.0), 50 mM KCl and 10 mM DTT. The protein concentrations were adjusted to 1.0 mM. The sample temperature was set at 310 K (37 °C) for all NMR experiments, unless otherwise noted. Data processing and analysis were performed with the programs NMRPipe [40] and KUJIRA [41] running with NMRview [42], respectively. The CYANA utility was used for automatic NOE assignments [43,44]. Backbone dihedral angle restraints were generated by TALOS+ [45]. The 50 lowest-target function CYANA structures were subjected to explicit water refinement using the program XPLOR-NIH with distance and dihedral restraints [46]. The 10 lowest-energy structures calculated by XPLOR-NIH were validated using the program PROCHECK-NMR [47] and PSVS [48]. The structural statistics for SBD(∆CDE) and SBD(∆CDE)-y are summarized in Table S1. The programs PyMOL (DeLano Scientific, San Carlos, CA, USA) and MOLMOL [49] were used for structural analyses and figure generation. The resonance assignments and structural data for SBD(∆CDE) and SBD(∆CDE)-y have been deposited in the BMRB and PDB databases, respectively. BMRB accession codes: 36077 and 36078; and PDB entries: 5XI9 and 5XIR. 

### 4.3. NMR Spin Relaxation Experiments

All backbone ^15^N *R*_1_ and *R*_2_ relaxation rates and steady state heteronuclear ^15^N NOE (hNOE) data were collected on the 700 MHz NMR spectrometer at 298 K (25 °C) [39]. Peak intensities were measured by averaging over the signal intensities at the peak center and its eight surrounding points (nine-point averaging). Each peak center was found by the SPARKY “pc” function (T.D. Goddard and D. G. Kneller, SPARKY 3, University of California, San Francisco, CA, USA). The delays for *R*_1_ measurements (*t*_relax_) were 10.3 (twice), 153.9, 307.9, 461.8, 615.7 (twice), 769.6, 923.6, 1128.8 and 1539.3 ms, whereas the delays for *R*_2_ (*t*_relax_) were 0.0, 16.0 (twice), 40.0, 80.0 (twice) and 160.0 ms. The spectra for *R*_1_ and *R*_2_ were collected in an interleaved manner. For measuring hNOEs, paired spectra were recorded in an interleaved manner in which ^1^H saturation of 3 s was applied alternatively with the recycle delay set to 2 s. *R*_1_ and *R*_2_ relaxation rate constants for each signal were determined by fitting the data using the modelXY TCL built-in function of NMRPipe [40]. Uncertainties for *R*_1_ and *R*_2_ were estimated by a Monte Carlo approach using the duplicated data points. The uncertainty for each hNOE value was evaluated by using the standard deviation of the noise in a spectral region with no peaks, which was obtained by the NMRPipe built-in module.

### 4.4. Isothermal Titration Calorimetry (ITC) Experiments

ITC experiments consisted of a series of 1.5 μL injections of 5 mM NR-peptide (NRLLLTG) into a 200 μL SBD(∆CDE)-y sample solution (0.1 mM) in the thermostatic cell with an initial delay of 60 s, a 3 s injection period and a spacing between injections of 150 s using a Microcal Auto iTC200 instrument (Malvern, Worcestershire, UK). The protein concentration was determined by the absorption at 280 nm using a Nanodrop 2000 (Thermo Fisher Scientific, Waltham, MA, USA). The NR-peptide was weighed using an electronic microbalance CP225D (Sartorius, Lower Saxony, Germany). The collected data were analyzed with the Microcal ORIGIN software (Malvern). The corrected binding isotherms were fitted using a single-site with the stoichiometry fixed as *n* = 1, because the system in this study indicated low binding affinity [50,51]. The experiments were performed in triplicate. *K*_D_ and its error was determined as an average and a standard deviation of the *K*_D_ values determined in each of the three trials. 

### 4.5. NMR Titration Experiments

The NMR titration experiments were performed by acquiring a series of 2D ^1^H-^15^N HSQC spectra of the protein in 50 mM potassium phosphate (pH 7.0), 50 mM KCl and 10 mM DTT at 310 K (37 °C). The NR-peptide (0.075 mM, 0.15 mM, 0.30 mM, 0.60 mM, 1.2 mM, 3.0 mM and 6.0 mM) was titrated into a sample containing 0.3 mM ^15^N labeled SBD(∆CDE)-y. Data processing and analysis were performed with NMRPipe [40] and KUJIRA [41], respectively. The dissociation constant, *K*_D_, was estimated by fitting Equation (1) to the bound fraction of SBD(∆CDE)-y, *f*_bound_:

(1) $$\begin{array}{c} f_{\mathrm{bound}}\left({\left[L\right]}_{\mathrm{total}}\right)=1-\frac{I\left({\left[L\right]}_{\mathrm{total}}\right)}{I_{0}}\\ =\frac{\left({\left[P\right]}_{\mathrm{total}}+{\left[L\right]}_{\mathrm{total}}+K_{D}\right)-\sqrt{{\left({\left[P\right]}_{\mathrm{total}}+{\left[L\right]}_{\mathrm{total}}+K_{D}\right)}^{2}-4{\left[P\right]}_{\mathrm{total}}{\left[L\right]}_{\mathrm{total}}}}{2{\left[P\right]}_{\mathrm{total}}} \end{array}$$

where [*L*]_total_ and [*P*]_total_ are the total concentrations of NR-peptide and SBD(∆CDE)-y, respectively, and *I* and *I*_0_ are the signal intensities with and without NR-peptide. Each signal intensity was measured by a similar procedure described in Section 4.3. In estimating the *K*_D_ values, we simultaneously used the buildup profiles from the signals of 28 residues for fitting, as in a global fitting manner: the 28 residues were selected according to their spectral intensities *I*(1.2 mM) collected in the presence of 1.2 mM NR-peptide with the criterion *ratio* = *I*(1.2 mM)/*I*_0_ < *ratio*_ave_ − 0.8σ. Uncertainties in the dissociation constant was estimated by Monte Carlo simulations on the basis of the uncertainty for each signal intensity evaluated using the root-mean-square deviation value on a spectral region with no signals, which was obtained by the NMRPipe built-in module [40].

### 4.6. NMR Paramagnetic Relaxation Enhancement Experiments

We prepared the SBD(∆CDE) (S537C/L542Y) mutant as described in Section 4.1 to attach the nitroxide radical. The SBD(∆CDE) (S537C/L542Y) mutant was incubated with 10-fold molar excess of (1-oxyl-2,2,5,5-tetramethyl-delta(3)-pyrroline-3-methyl)-methanethiosulfonate (MTSL), or a diamagnetic control MTSL analog, (1-acetoxy-2,2,5,5-tetramethyl-δ-3-pyrroline-3-methyl)methane-thiosulfonate (Toronto Research Chemicals, Toronto, ON, Canada), dissolved in DMSO at 296 K (23 °C) for 10 h. The sample solution was dialyzed extensively against buffer E at 277 K (4 °C) to remove unreacted MTSL. To check the completion of the reaction, the dialyzed sample was denatured with 8 M urea and then incubated with 10-fold molar excess of PEG-maleimide (Nichiyu, Tokyo, Japan). The amount of non-reacted protein that was attached to PEG-maleimide was confirmed by SDS-PAGE. We collected 2D ^1^H-^15^N HSQC spectra for 0.5 mM SBD(∆CDE) (S537C/L542Y) mutant in 50 mM potassium phosphate (pH 7.0) and 50 mM KCl with/without 5.0 mM NR-peptide at 310 K (37 °C). Each peak intensity, *I*, was measured by the same procedure described in Section 4.3. PREs were estimated by PRE = *I*_with MTSL_/*I*_with MTSL analog_. The error estimation was evaluated using the standard deviation of the noise in a spectral region with no peaks, which was obtained by the NMRPipe built-in module.

## 5. Conclusions

By solving the solution structures of SBD(∆CDE) and SBD(∆CDE)-y we have revealed two aspects of the response of Hsp70-SBD upon binding to a client-peptide. The first aspect is that client-peptide binding allosterically rearranges residues in the lynchpin site of the interdomain contact between the SBD and NBD, as observed in DnaK [27,28]. Hsp70 SBD, however, responded to peptide binding differently from that observed in DnaK; the distance between the substrate binding loops L_1,2_ and L_3,4_ of Hsp70 increases upon peptide binding, whereas the corresponding loops in DnaK move closer to each other to grasp the substrate [27]. In Hsp70 SBD, the change in the conformation of L_1,2_ caused rearrangement of β2, which displaces R416 as one of the key residues interacting with NBD (Figure 8). The other key residue, N483, at this interface is displaced by the movement of L486 caused by peptide binding to the cleft, as similarly observed in DnaK [28]. The conformational change to the L_α__,β_ loop following peptide binding hinders the interaction of E444 and L456 with the NBD, which was also found in DnaK [27]. The second aspect revealed is the conformational change to the αLid upon client-peptide binding. Peptide binding may disrupt the interaction between R447 and D529 to release the αLid from βSBD, leading to this lid adopting a more dynamic state (Figure 5, Figure 6 and Figure S10). The change in the dynamics of the αLid induced by client-peptide binding should facilitate αLid binding to NBD in the ATP bound form [18]. 

Taken together, this work shows that both Hsp70 and DnaK adopt a similar interdomain communication mechanism through structural rearrangement of the lynchpin site for interdomain contact, although intradomain allostery within βSBD happens in a manner slightly different from that observed in DnaK. Hsp70 SBD is highly homologous to DnaK SBD (47% sequence identity, Figure S1), and the structures of the SBDs from both species are similar [15]. Nonetheless, sequence variations likely explain the different allosteric responses upon substrate binding. The present comparative study regarding interdomain communication of Hsp70 will deepen our understanding of how this family of chaperones function.

## Supplementary Materials

The following are available online, Figure S1: Sequence alignment of human Hsp70 (HSPA1A) and bacterial (*E. coli*) DnaK protein sequences; Figure S2: Structural changes caused by elimination of the intramolecular interaction between αB in αLid and βSBD; Figure S3: Structural flexibility in SBD(∆CDE) and SBD(∆CDE)-y; Figure S4: Structural comparison of the substrate binding loops, L_1,2_ and L_3,4_; Figure S5: Difference in the conformation of the L_α,β_ loop between SBD(∆CDE) and SBD(∆CDE)-y; Figure S6: Normalized weighted average ^1^H and ^15^N chemical shift differences between SBD(∆CDE) and SBD(∆CDE)-y; Figure S7: Isothermal titration calorimetry data for the NR-peptide titration with SBD(∆CDE)-y; Figure S8: Change in the PRE effects caused by NR-peptide binding to SBD(∆CDE)-y; Figure S9: Changes in signal intensities caused by the NR-peptide binding to SBD(∆CDE)-y; Figure S10: Inter-subdomain interaction mediated by salt bridge between R447 and D529; Figure S11: Comparison of the positions of residues R447 and D529 engaged in the inter-subdomain interaction; Figure S12: Comparison of the positions of residues engaged in the interdomain contact between SBD and NBD; and Table S1: Structural statistics for the 10 lowest-energy structures of SBD(∆CDE) and SBD(∆CDE)-y.
