# Supplementary material for: Substrate Binding Switches the Conformation at the Lynchpin Site in the Substrate-Binding Domain of Human Hsp70 to Enable Allosteric Interdomain Communication
Source: Molecules. 2018 Feb 27;23(3):528. doi: 10.3390/molecules23030528 (PMC6017645; doi:10.3390/molecules23030528)
Supplement: Supplementary file 1 [file molecules-23-00528-s001.pdf]

Supplementary Materials

Substrate Binding Switches the Conformation at the Lynchpin Site in the Substrate-Binding Domain of Human Hsp70 to Enable Allosteric Interdomain Communication

Kohei Umehara [1,†], Miho Hoshikawa [1,†], Naoya Tochio [2,‡], and Shin-ichi Tate [1,2,*]

[1] Department of Mathematical and Life Sciences, School of Science, Hiroshima University, 1-3-1 Kagamiyama, Higashi-Hiroshima 739-8526, Japan; m161409@hiroshima-u.ac.jp (K.U.); miho.hoshikawa.0925@gmail.com (M.H.)

[2] Research Center for the Mathematics on Chromatin Live Dynamics (RcMcD), Hiroshima University, 1-3-1 Kagamiyama, Higashi-Hiroshima 739-8526, Japan; naoya-tochio@hiroshima-u.ac.jp (N.T.)

‡ Present address: Faculty of Pharma-Sciences, Teikyo University, 2-11-1, Kaga, Itabashi-ku, Tokyo 173-8605, Japan; tochio@pharm.teikyo-u.ac.jp (N.T.)

† These authors equally contributed to this work

* Correspondence: tate@hiroshima-u.ac.jp; Tel.: +81-82-424-7387

Supplementary Figures: Figure S1 – S12

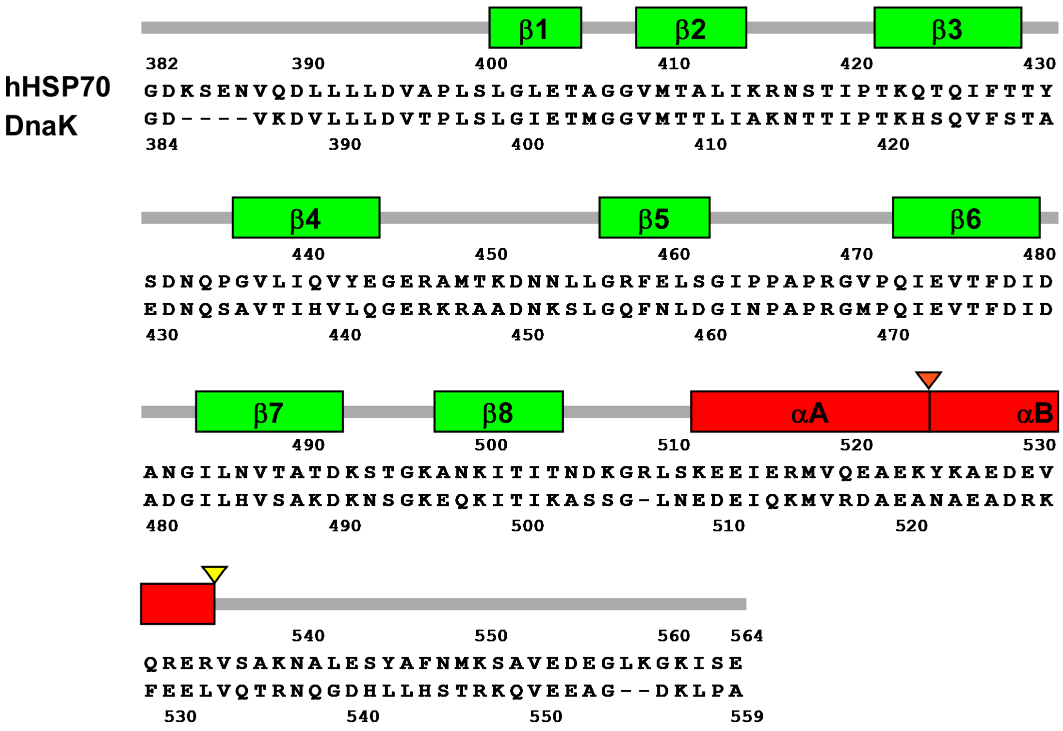

Figure S1. Sequence alignment of human Hsp70 (HSPA1A) and bacterial (*E. coli*) DnaK protein sequences. Secondary structure elements determined for SBD(ΔCDE) by NMR are shown with boxes in green (β-strand) and red (α-helix). The construct for SBD(ΔCDE) contains the sequence (residues 382–564) shown for hHsp70. The red triangle is the hinge position between αA and αB in the αLid. The junction position at which the α-helical stability changes in αB of the αLid is indicated by the yellow triangle.

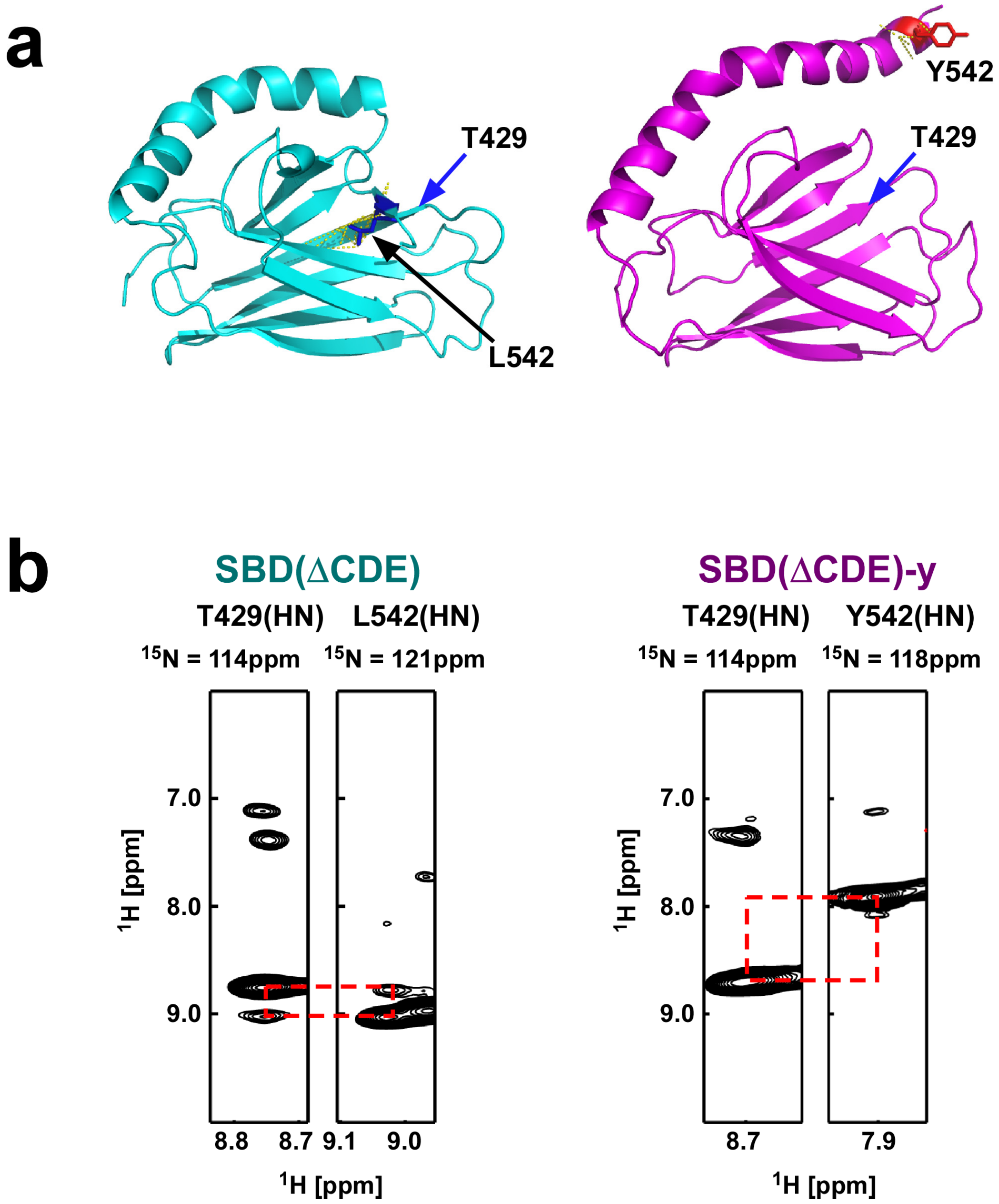
a
T429
L542
Y542
T429
b
SBD(ΔCDE)
T429(HN)
L542(HN)
^{15}N = 114ppm
^{15}N = 121ppm
SBD(ΔCDE)-y
T429(HN)
Y542(HN)
^{15}N = 114ppm
^{15}N = 118ppm
7.0
8.0
9.0
^{1}H [ppm]
8.8
8.7
9.1
9.0
8.7
7.9
^{1}H [ppm]

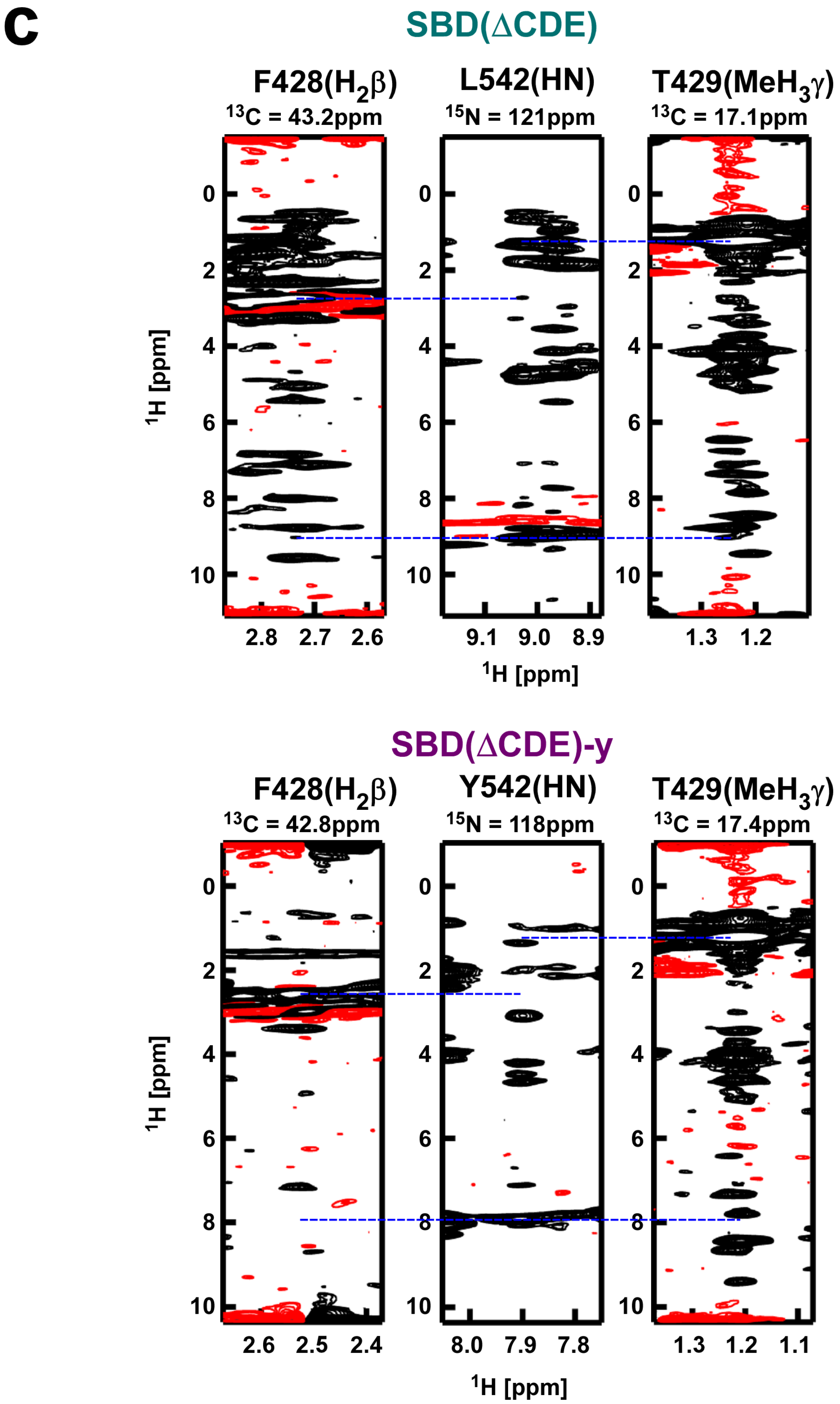
c
SBD(ΔCDE)
F428(H2β)
13C = 43.2ppm
L542(HN)
15N = 121ppm
T429(MeH3γ)
13C = 17.1ppm
1H [ppm]
0
2
4
6
8
10
2.8 2.7 2.6
9.1 9.0 8.9
1.3 1.2
SBD(ΔCDE)-y
F428(H2β)
13C = 42.8ppm
Y542(HN)
15N = 118ppm
T429(MeH3γ)
13C = 17.4ppm
2.6 2.5 2.4
8.0 7.9 7.8
1.3 1.2 1.1

Figure S2. Structural changes caused by elimination of the intramolecular interaction between αB in αLid and βSBD. (a) Structures of SBD(ΔCDE) and SBD(ΔCDE)-y showing the observed NOEs (yellow dashed lines) for T429/L542 and T429/Y542, respectively. (b) Changes in the NOE pattern between the amide protons of the residues T429 and L(Y)542 because of the L542Y mutation. (c) The additional NOEs to connect L542 to F428 and T429 are lost in the L542Y mutant: $H_2\beta$ and $MeH_3\gamma$ denote unresolved β methylene protons and γ methyl protons for F428 and T429, respectively. The mutation removes the intramolecular interaction between αB and the substrate binding cleft in βSBD. The signals in red represent the negative intensities due to the spectral folding over in ^{13}C dimension.

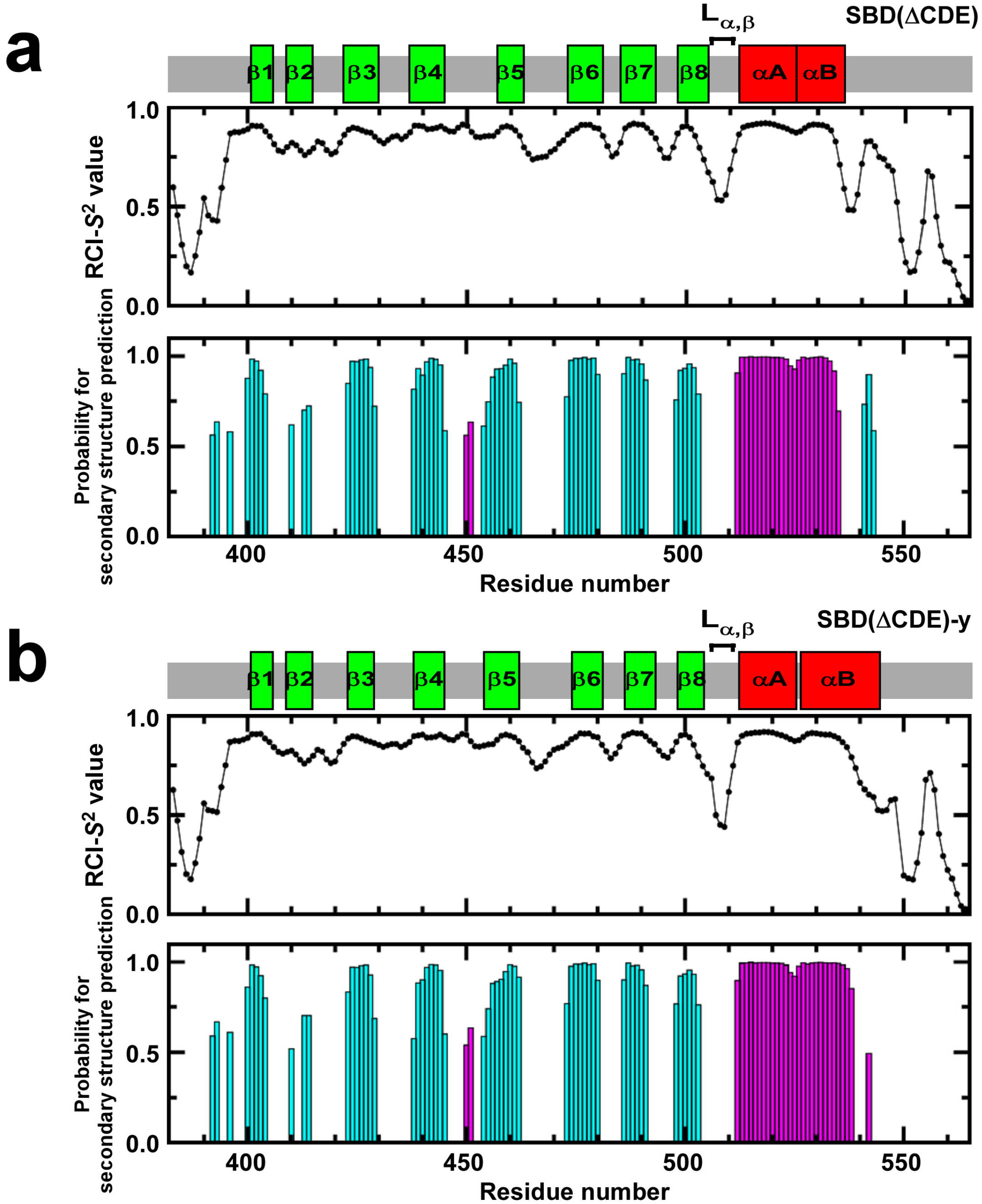
a
SBD(ΔCDE)
L α,β
β1 β2 β3 β4 β5 β6 β7 β8 αA αB
RCI-S2 value
Probability for secondary structure prediction
Residue number
b
SBD(ΔCDE)-y

c

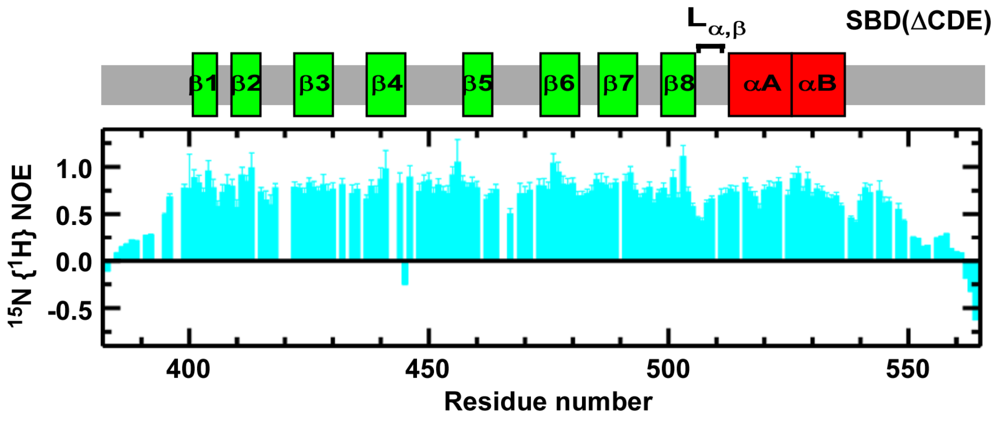

d

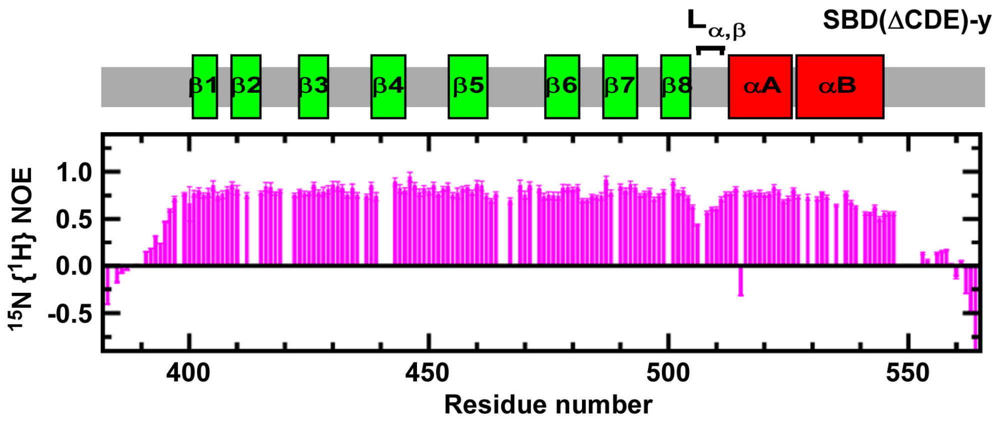

Figure S3. Structural flexibility in SBD(ΔCDE) and SBD(ΔCDE)-y. Predicted secondary structures from the backbone chemical shifts using the programs RCI (top) [1] and TALOS+ (bottom) [2] for (a) SBD(ΔCDE) and (b) SBD(ΔCDE)-y. (c) the hNOE values for SBD(ΔCDE) (cyan) and (d) SBD(ΔCDE)-y (magenta)

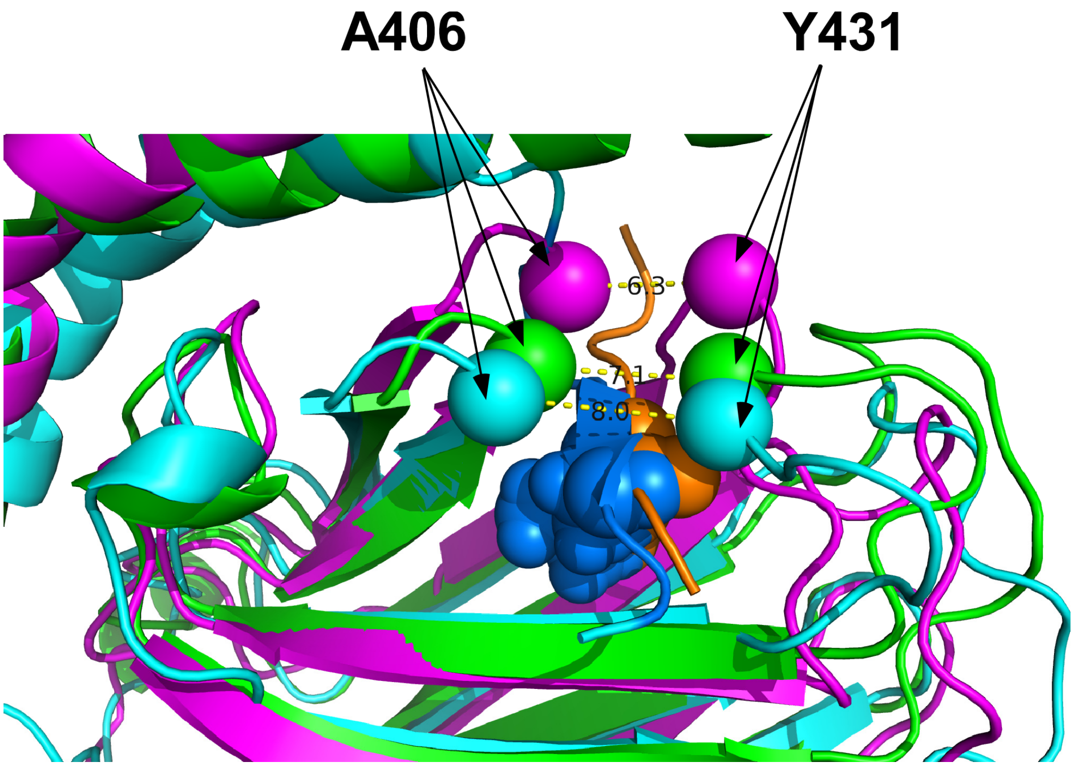

Figure S4. Structural comparison of the substrate binding loops, $L_{1,2}$ and $L_{3,4}$. Superpositioned structures of SBD(ΔCDE)-y (magenta), SBD(ΔCDE) (cyan) and the crystal structure of the NR-peptide bound SBD of human Hsp70 (green) (PDB ID: 4PO2) [3]. The Cα positions of A406 ($L_{1,2}$) and Y431 ($L_{3,4}$) are drawn as spheres. The distances between the Cα atoms of A406 and Y431 for the three structures are presented in yellow.

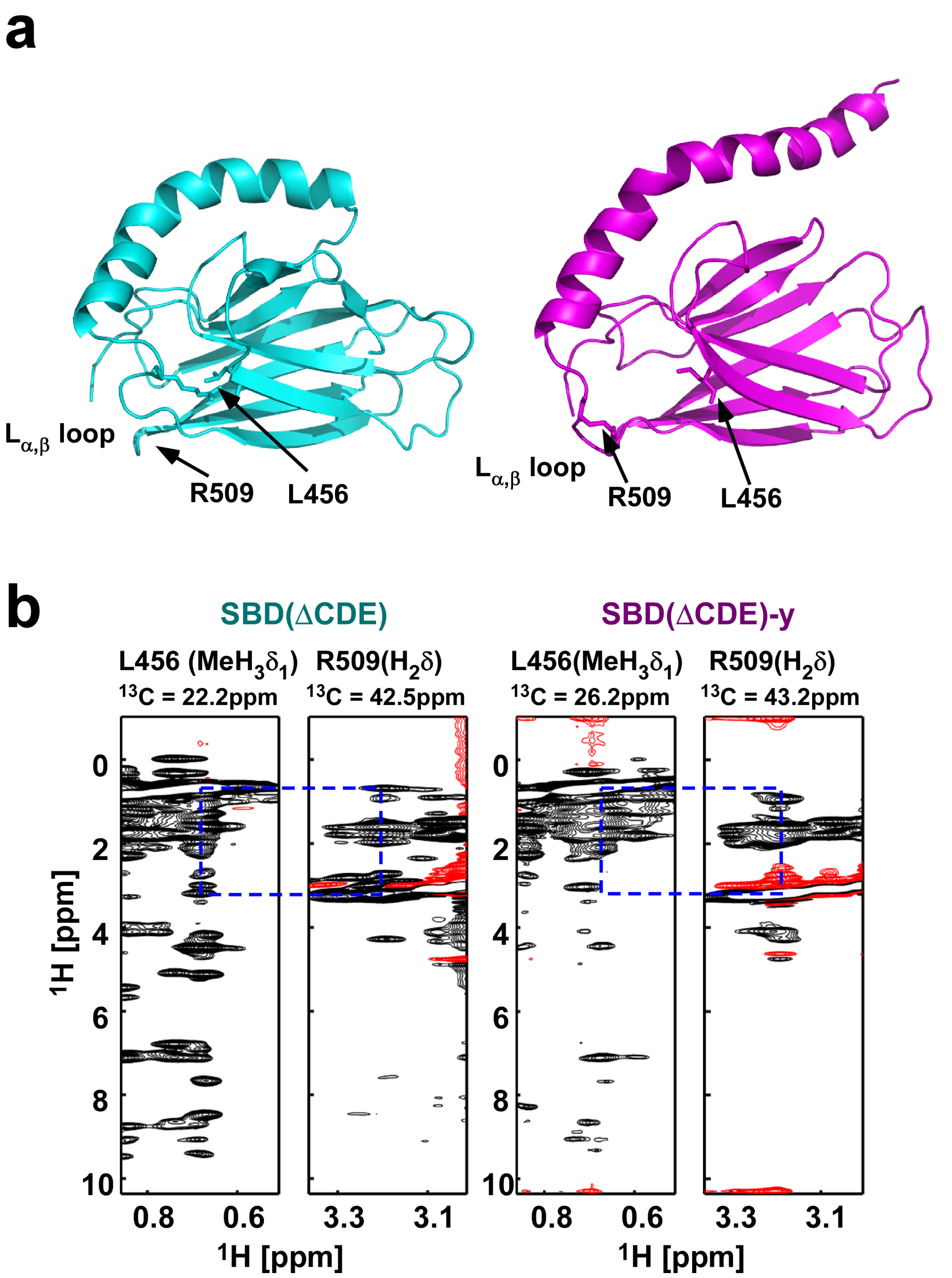

Figure S5. Difference in the conformation of the $L_{\alpha,\beta}$ loop between SBD(ΔCDE) and SBD(ΔCDE)-y. (a) Structures of SBD(ΔCDE) and SBD(ΔCDE)-y with the side chains of residues L456 and R509 shown as stick models. (b) The NOEs observed for residues L456 and R509 in SBD(ΔCDE) are absent in the corresponding spectrum for SBD(ΔCDE)-y.

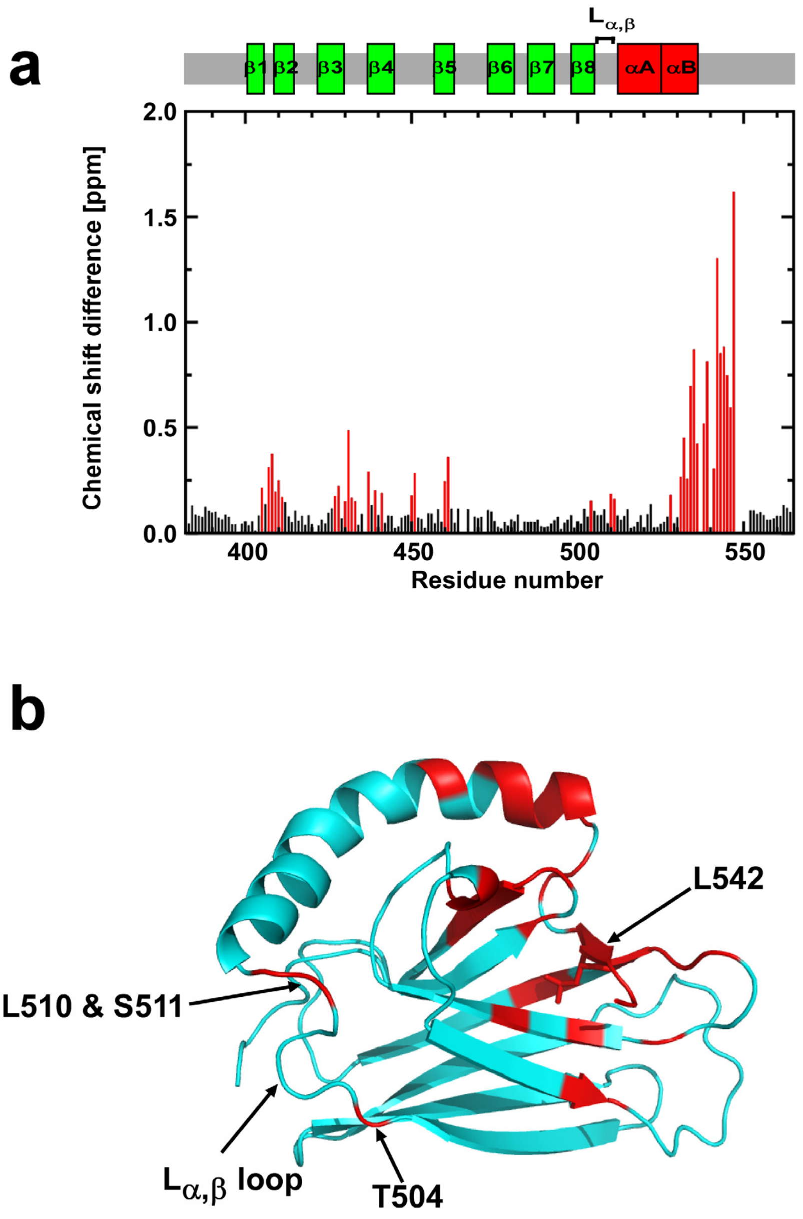

Figure S6. Normalized weighted average ^{1}H and ^{15}N chemical shift differences between SBD(ΔCDE) and SBD(ΔCDE)-y. (a) Per residue weighted chemical shift differences, $\Delta\delta = \sqrt{\Delta\delta(^{1}\mathrm{H})^{2} + \left(\Delta\delta(^{15}\mathrm{N})/5\right)^{2}}$, are plotted. The $\Delta\delta$ values that are above the average value (0.147ppm) are shown in red and (b) have been mapped onto the SBD(ΔCDE) structure (red).

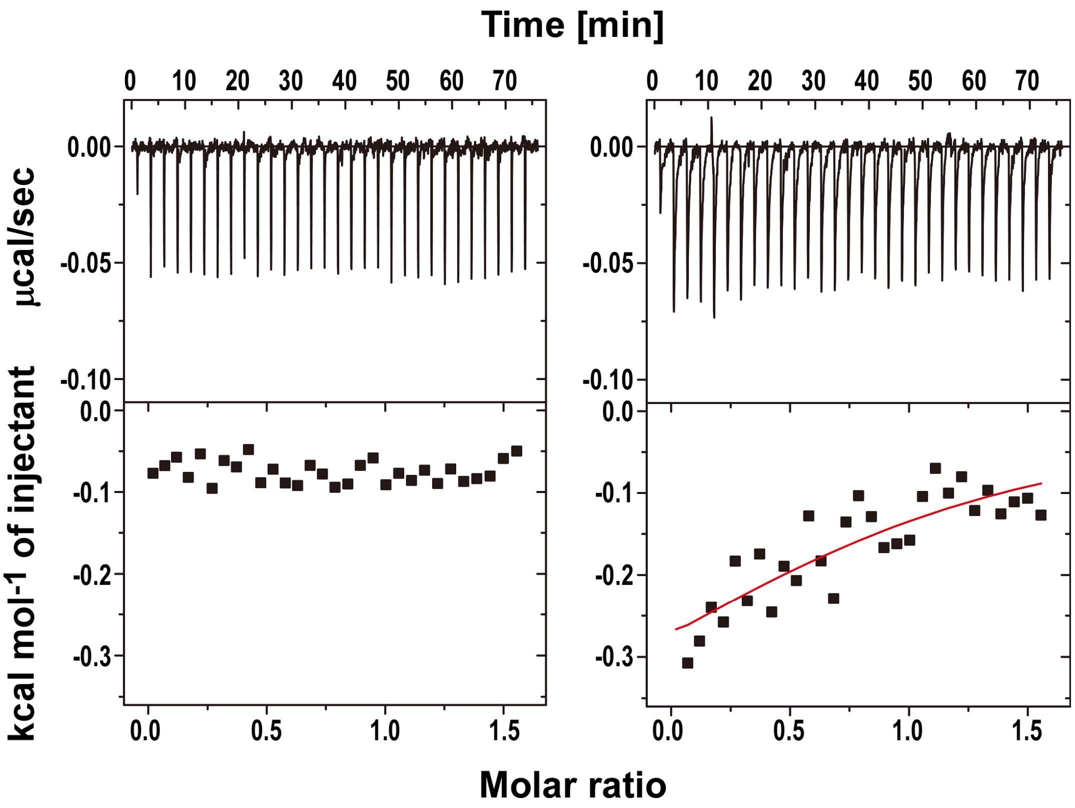

Figure S7. Isothermal titration calorimetry data for the NR-peptide titration with SBD(ΔCDE)-y. Each thermal response comes from 1.5 μL injections of the peptide containing solution to the buffer solution (left) or to the SBD(ΔCDE)-y solution (right). The thermogram coupled with the peptide binding (right) is significantly greater in magnitude than that observed in dilution (left). The solid red line in the ΔH plot is the best-fit model function assuming a 1:1 stoichiometry between the peptide and SBD(ΔCDE)-y. Triplicated ITC data were subjected to the fitting with the model function to give a K_D = 1.5 ± 0.3 mM, as an average and standard deviation of the three K_D values determined in each experiment.

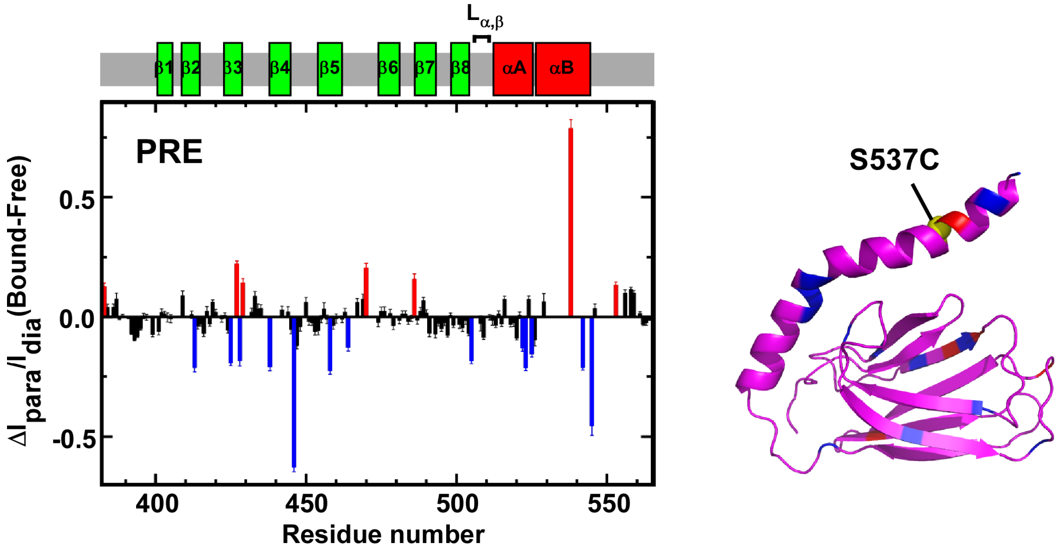

Figure S8. Change in the PRE effects caused by NR-peptide binding to SBD(ΔCDE)-y. The PRE effect was evaluated as the NMR intensity ratio I_{para}/I_{dia}, where I_{para} and I_{dia} denote the NMR signal intensities collected for the protein labeled with the paramagnetic spin label and the diamagnetic label, respectively. The graph shows the per residue difference in the PRE effect ($\Delta I_{para}/I_{dia}$) between the presence and absence of the NR-peptide, where $\Delta I_{para}/I_{dia} = I_{para}/I_{dia}$ (peptide-bound) – I_{para}/I_{dia} (free). Red and blue bars represent $\Delta I_{para}/I_{dia}$ values for residues that are greater than one standard deviation (0.107 as an absolute value) above the average $\Delta I_{para}/I_{dia}$ value (0.072 as an absolute value). Positive $\Delta I_{para}/I_{dia}$ values (red) indicate that residues have moved away from the spin label at residue 537 upon SBD(ΔCDE)-y binding the peptide, whereas negative $\Delta I_{para}/I_{dia}$ values (blue) indicate that residues have moved closer to the spin label in the peptide-bound state.

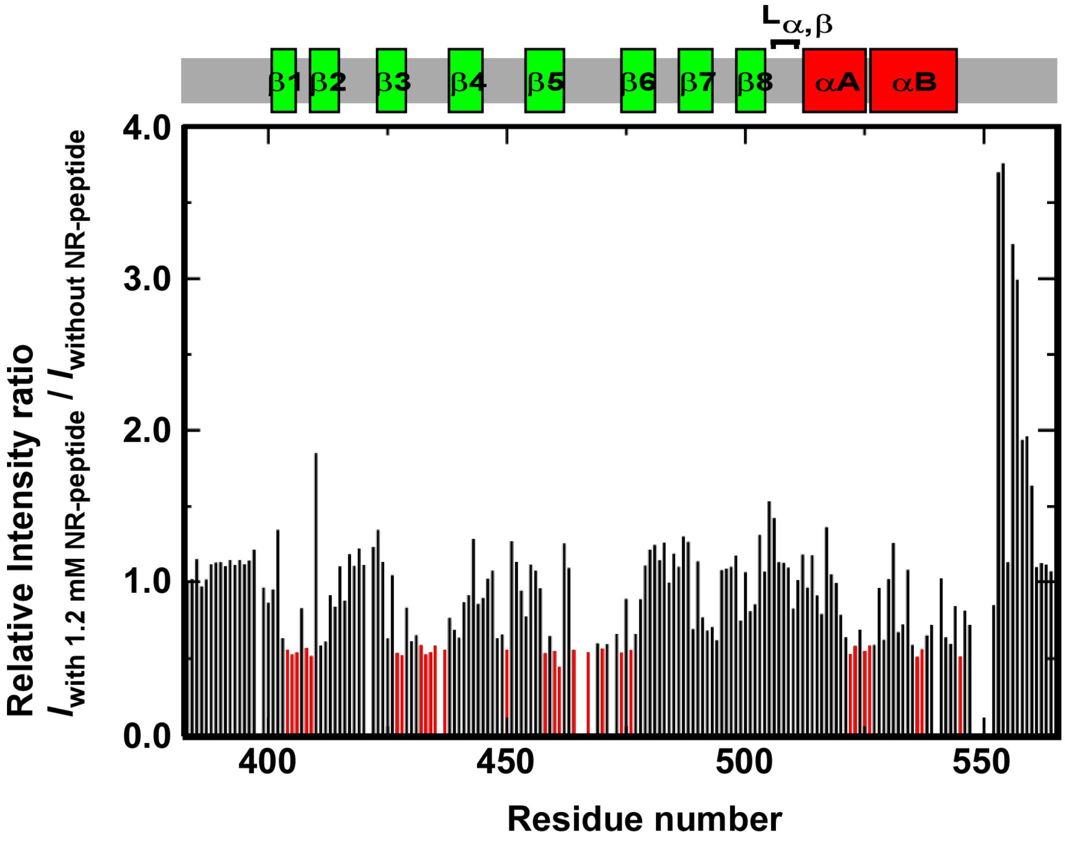

Figure S9. Changes in signal intensities caused by the NR-peptide binding to SBD(ΔCDE)-y. Binding of the NR-peptide to SBD(ΔCDE)-y caused NMR spectral changes in the slow exchange time regime on the chemical shift time scale. Peptide-binding to SBD(ΔCDE)-y was, therefore, monitored by the reduction in the intensities of the resonances for the free protein state. The relative NMR signal intensities collected for the protein in the presence of the NR-peptide at 1.2 mM concentration against the corresponding signals for the free-state protein (0.3 mM) are plotted. Values less than 0.8 times the standard deviation (0.386) from the average value (0.970) are marked in red, showing that the corresponding residues are engaged in peptide binding when compared with that of the other residues in the protein.

a

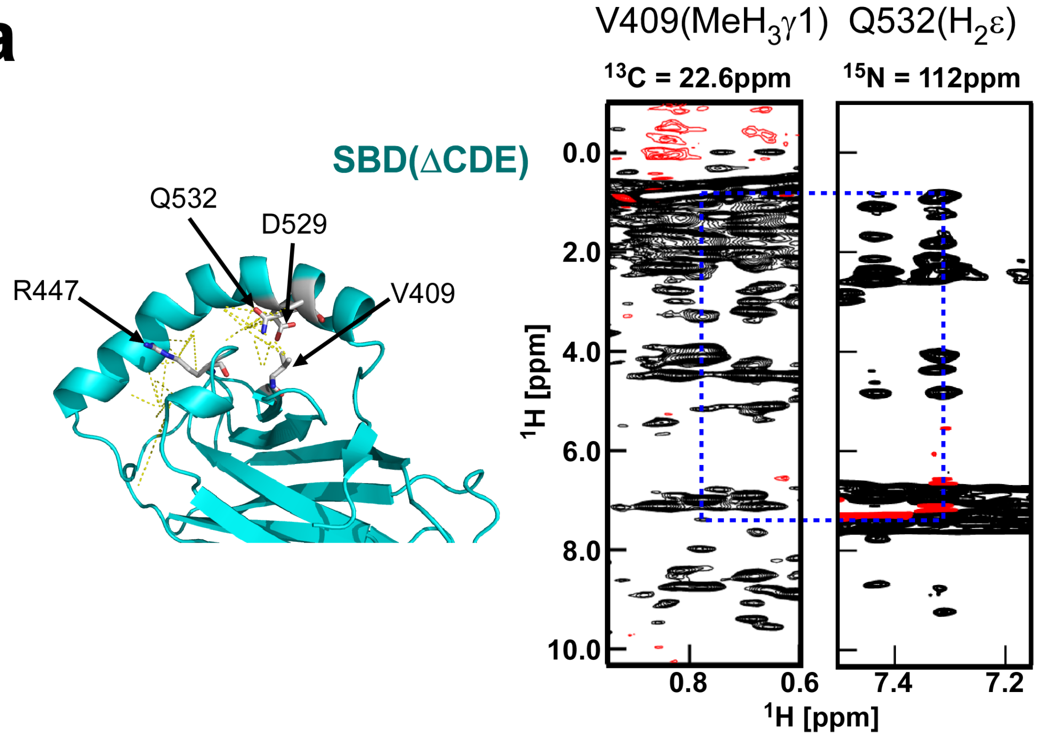

SBD(ΔCDE)
Q532
D529
R447
V409
V409(MeH3γ1)
Q532(H2ε)
13C = 22.6ppm
15N = 112ppm
0.0
2.0
4.0
6.0
8.0
10.0
1H [ppm]
0.8
0.6
7.4
7.2
1H [ppm]

b

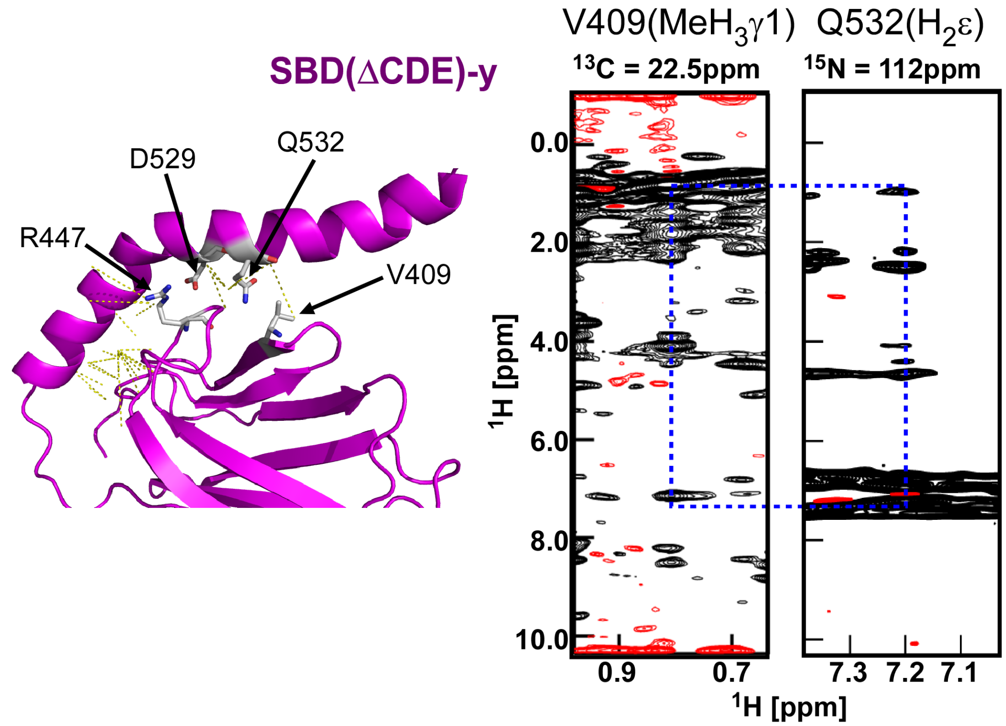

SBD(ΔCDE)-y
D529
Q532
R447
V409
V409(MeH3γ1)
Q532(H2ε)
13C = 22.5ppm
15N = 112ppm
0.0
2.0
4.0
6.0
8.0
10.0
1H [ppm]
0.9
0.7
7.3
7.2
7.1
1H [ppm]

Figure S10. Inter-subdomain interaction mediated by salt bridge between R447 and D529. (a) NOEs observed between the αLid and βSBD in SBD(ΔCDE) are depicted as yellow dashed lines (right) with the residue positions for R447 and Q532 drawn in ball-and-stick representation. (b) NOEs observed between the αLid and βSBD in SBD(ΔCDE)-y are depicted as yellow dashed lines The NOE between V409 and Q532 was absent in SBD(ΔCDE)-y, indicating that the αB location differs from that in SBD(ΔCDE). The signals in red in ^{13}C 3D-NOESY spectrum are negative intensities due to the spectral folding along ^{13}C dimension.

a

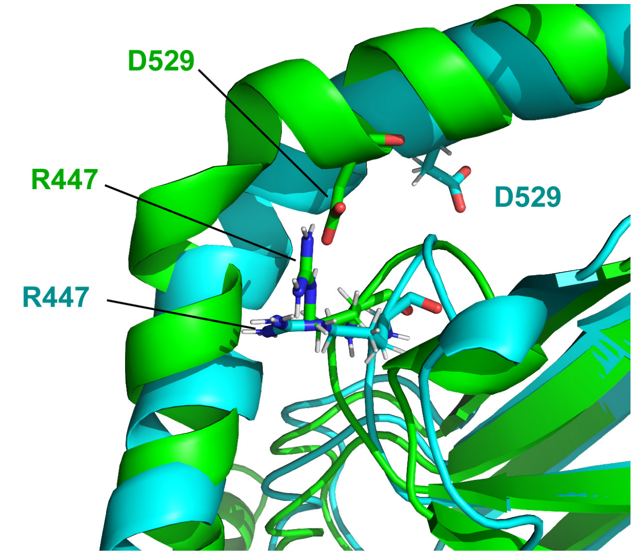

b

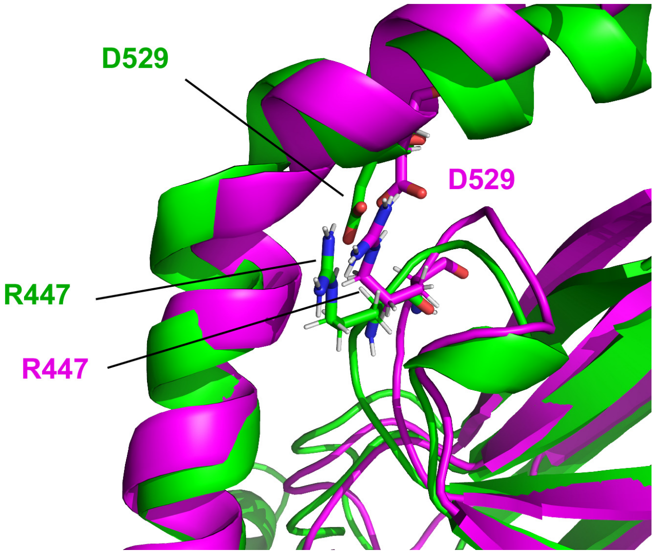

Figure S11. Comparison of the positions of residues R447 and D529 engaged in the inter-subdomain interaction. (a) Superposed representation of the inter-subdomain contact site of SBD(ΔCDE) (cyan) and the crystal structure of the substrate-bound Hsp70 SBD (green) (PDB ID: 4PO2) [3]. (b) Structural comparison between SBD(ΔCDE)-y (magenta) and the crystal structure of the substrate-bound Hsp70 SBD (green).

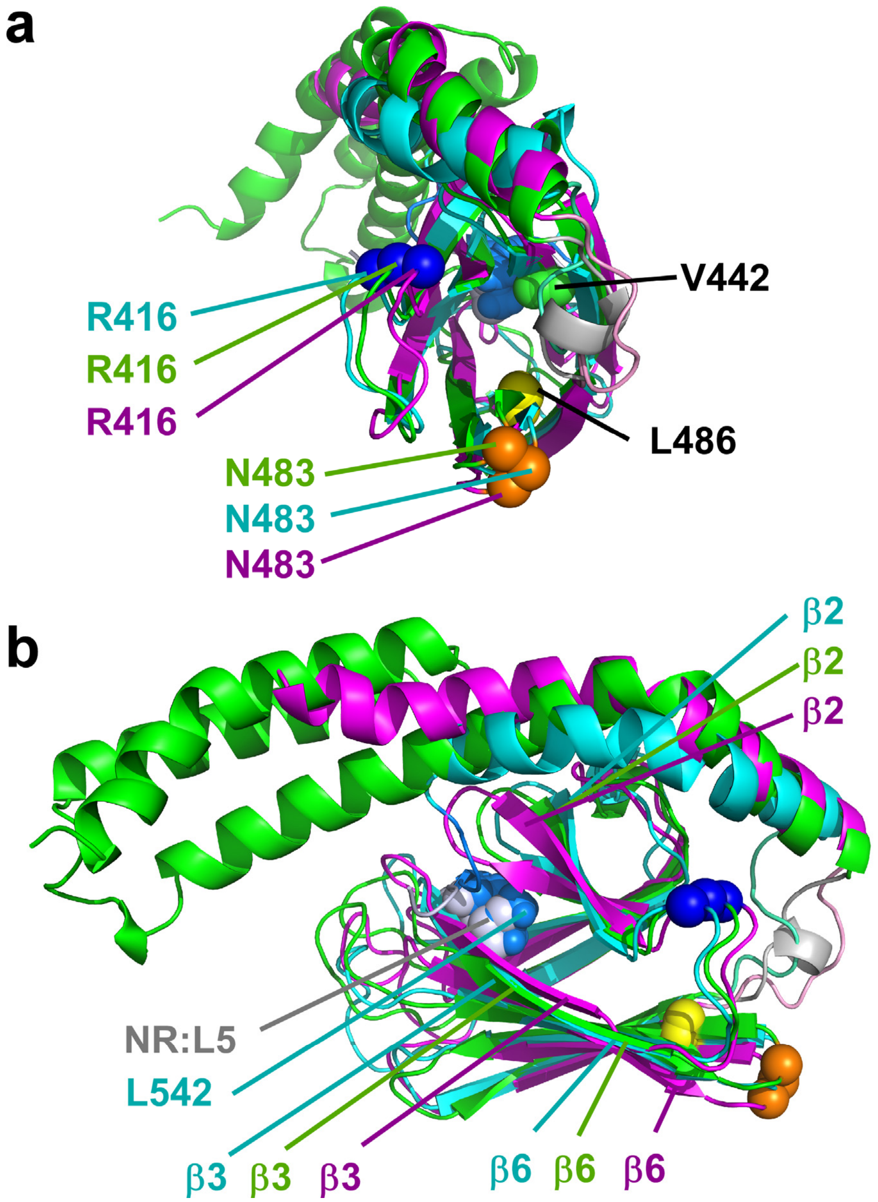

Figure S12. Comparison of the positions of residues engaged in the interdomain contact between SBD and NBD. (a) The interdomain contact site structures of SBD(ΔCDE) (cyan), SBD(ΔCDE)-y (magenta) and the crystal structure of the substrate-bound Hsp70 SBD (PDB ID: 4PO2) [3] are superpositioned. Cα positions of R416 and N483 are represented by spheres. (b) The same structural superposition of the three proteins displayed at a different orientation. L542 (light blue) in the substrate binding cleft of SBD(ΔCDE) and L5 (gray) of the NR-peptide in the Hsp70 SBD crystal structure are shown as spheres.

Supplementary Table

Table S1. Structural statistics of the 10 lowest-energy structures of SBD(ΔCDE) and SBD(ΔCDE)-y.

	SBD(ΔCDE)	SBD(ΔCDE)-y
Completeness of resonance assignments (%)[a]		
Backbone	95.8	92.1
Side chain	90.6	86.9
Aromatic	78.9	95.2
Stereospecific methyl	87.9	79.2
Conformational restraints		
Distance restraints		
Total	2,208	1,828
Intraresidue ($i = j$)	592	569
Sequential ($\|i-j\| = 1$)	662	550
Medium range ($1 < \|i-j\| < 5$)	269	216
Long range ($\|i-j\| \geq 5$)	685	493
Dihedral angle restraints[b]	202	226
No. of restraints per residue	11.9	9.9
No. of long-range restraints per residue	3.7	2.7
Residual restraint violations		
Average no. of distance violations per structure		
0.1−0.3 Å	2.8	2.0
0.3−0.5 Å	0.5	0.5
> 0.5 Å	0.0	0
Average no. of dihedral angle violations per structure		
> 5°	0.0	0
Model quality[c]		
RMSD backbone atoms (Å)[d]	1.21 ± 0.24	1.22 ± 0.29 (2.43 ± 0.91)
RMSD heavy atoms (Å)[d]	1.83 ± 0.30	1.78 ± 0.33 (2.98 ± 0.88)
RMSD bond lengths (Å)	0.008	0.008
RMSD bond angles (°)	0.6	0.6
MolProbity Ramachandran statistics[c]		
Most favored regions (%)	96.6[e]	96.7[f]
Allowed regions (%)	3.3[e]	3.0[f]
Disallowed regions (%)	0.1[e]	0.3[f]
Global quality scores (raw / Z score)[c]		
Verify3D	0.32 / –2.25	0.27 / –3.05
Prosall	0.27 / –1.57	0.31 / –1.41
PROCHECK (ϕ-ψ)	–0.45 / –1.46[e]	–0.50 / –1.65[f]
PROCHECK (all)	–0.42 / –2.48[e]	–0.44 / –2.60[f]
MolProbity clash score	16.51 / –1.31	14.51 / –0.96
Model contents		
Total no. of residues	185	185
BMRB accession number	36077	36078
PDB ID code	5XI9	5XIR

[a]The number excluding highly exchangeable protons, nitrogens bound with highly exchangeable protons, nonprotonated carbons and nitrogens.
[b]The angle restraints were derived from TALOS+ [2] with the angle ranges ± 30°.
[c]Calculated using PSVS version 1.5 [4]. The steric clash noted in the significant value in Disallowed regions may come from the ambiguous puckers of Pro residues. In the present calculation, all Pro residues were fixed to C^{γ}-endo, although some Pro may interconvert between C^{γ}-exo and C^{γ}-endo. In the present structures, we realized Pro472 in $L_{5,6}$ loop tends to cause steric clash, presumably due to some extent of the population of C^{γ}-exo pucker occurring in Pro472.
[d]For residues in secondary structure elements (L401–T405, V409–I414, T422–T430, G437–E444, T450–D452, N454–S462, I474–I480, L486–D492, A498–I503 and K512–K524). In parentheses for SBD(ΔCDE)-y, the RMSD values take into account the non-converged αB (K526–E543).
[e]For residues L393–R416, S418–S432, V438–E444, A448–L456, R458–S462, I464–P468, P472–T504, R509–R535 and A541–

E543, as the ordered residues estimated by PSVS.
[f]For residues V396–P436, V438–S462, I464–A467, Q473–T504 and R509–S544, as the ordered residues estimated by PSVS.

Supplementary References

1. Berjanskii, M.V.; Wishart, D.S. A simple method to predict protein flexibility using secondary chemical shifts. *Journal of the American Chemical Society* **2005**, *127*, 14970-14971.
2. Shen, Y.; Delaglio, F.; Cornilescu, G.; Bax, A. Talos+: A hybrid method for predicting protein backbone torsion angles from nmr chemical shifts. *Journal of biomolecular NMR* **2009**, *44*, 213-223.
3. Zhang, P.; Leu, J.I.; Murphy, M.E.; George, D.L.; Marmorstein, R. Crystal structure of the stress-inducible human heat shock protein 70 substrate-binding domain in complex with peptide substrate. *PloS one* **2014**, *9*, e103518.
4. Bhattacharya, A.; Tejero, R.; Montelione, G.T. Evaluating protein structures determined by structural genomics consortia. *Proteins* **2007**, *66*, 778-795.
